# SARS-CoV-2 Nsp5 Demonstrates Two Distinct Mechanisms Targeting RIG-I and MAVS To Evade the Innate Immune Response

**DOI:** 10.1128/mBio.02335-21

**Published:** 2021-09-21

**Authors:** Yongzhen Liu, Chao Qin, Youliang Rao, Chau Ngo, Joshua J. Feng, Jun Zhao, Shu Zhang, Ting-Yu Wang, Jessica Carriere, Ali Can Savas, Mehrnaz Zarinfar, Stephanie Rice, Hanging Yang, Weiming Yuan, Julio A. Camarero, Jianhua Yu, Xiaojiang S. Chen, Chao Zhang, Pinghui Feng

**Affiliations:** a Section of Infection and Immunity, Herman Ostrow School of Dentistry, Norris Comprehensive Cancer Center, University of Southern California, Los Angeles, California, USA; b Department of Chemistry, Dornsife College of Arts, Letters, and Sciences, University of Southern California, Los Angeles, California, USA; c Florida Research and Innovation Center, Cleveland Clinic, Port St. Lucie, Florida, USA; d Molecular and Computational Biology Section, University of Southern California, Los Angeles, California, USA; e Department of Molecular Microbiology and Immunology, Keck School of Medicine, Los Angeles, California, USA; f Department of Pharmaceutical Sciences, School of Pharmacy, University of Southern California, Los Angeles, California, USA; g Department of Hematology & Hematopoietic Cell Transplantation, City of Hope, Duarte, California, USA; Fujian Medical University

**Keywords:** E3 ligase, MAVS, Nsp5, RIG-I, SARS-CoV-2, protease, small-molecule inhibitor

## Abstract

Newly emerged severe acute respiratory syndrome coronavirus 2 (SARS-CoV-2) caused a global pandemic with astonishing mortality and morbidity. The high replication and transmission of SARS-CoV-2 are remarkably distinct from those of previous closely related coronaviruses, and the underlying molecular mechanisms remain unclear. The innate immune defense is a physical barrier that restricts viral replication. We report here that the SARS-CoV-2 Nsp5 main protease targets RIG-I and mitochondrial antiviral signaling (MAVS) protein via two distinct mechanisms for inhibition. Specifically, Nsp5 cleaves off the 10 most-N-terminal amino acids from RIG-I and deprives it of the ability to activate MAVS, whereas Nsp5 promotes the ubiquitination and proteosome-mediated degradation of MAVS. As such, Nsp5 potently inhibits interferon (IFN) induction by double-stranded RNA (dsRNA) in an enzyme-dependent manner. A synthetic small-molecule inhibitor blunts the Nsp5-mediated destruction of cellular RIG-I and MAVS and processing of SARS-CoV-2 nonstructural proteins, thus restoring the innate immune response and impeding SARS-CoV-2 replication. This work offers new insight into the immune evasion strategy of SARS-CoV-2 and provides a potential antiviral agent to treat CoV disease 2019 (COVID-19) patients.

## INTRODUCTION

A new coronavirus, known as severe acute respiratory syndrome coronavirus 2 (SARS-CoV-2), was reported in late 2019 and caused an ongoing worldwide pandemic (1, 2). As of 15 June 2021, SARS-CoV-2 was responsible for more than 178 million documented infections and 3.8 million deaths, in addition to many undetected asymptomatic infections (https://covid19.who.int). Vaccines against SARS-CoV-2 have been successfully developed within an unprecedented time frame of unprecedented record and are being administered under emergency authorization (3–6). However, antiviral therapy against SARS-CoV-2 is very limited (7–11). With the daily massive increase in infections and rapid emergency of immune-resistant mutants of SARS-CoV-2, effective antiviral therapy is desperately needed, (12–14). In general, antivirals slow down viral replication and assist the host immune response to defeat viral pathogens. How SARS-CoV-2 interacts with the human immune response is not well understood, which hampers the development of antiviral therapy.

Nucleic acids represent one classic molecular pattern associated with pathogens (15). SARS-CoV-2 is a positive, single-stranded RNA virus that generates double-stranded RNA (dsRNA) during replication (16). Previous studies have shown that SARS-CoV-2 induces a weak innate immune response in systems, including cultured cells, model animals, and patient samples (17–19). SARS-CoV-2 replicates within the cytoplasm of infected cells, although it compartmentalizes some replication processes within membrane-bound vesicles (16). RIG-I-like receptors, primarily RIG-I and MDA5, recognize the dsRNA structure to induce the activation of nuclear factor κB (NF-κB) and interferon (IFN) regulatory factor 3 (IRF3) (20–23). Along with other transcription factors, NF-κB and IRF3 choreograph the transcription of diverse inflammatory genes, establishing a potent innate antiviral immune response (24, 25). Among the inflammatory factors, interferons and other cytokines further amplify the host immune defense via inducing the expression of interferon-stimulated genes (ISGs) and other antiviral molecules, culminating in establishing potent antiviral immunity (26).

Previous studies on SARS-CoV and Middle East respiratory syndrome CoV (MERS-CoV) have identified diverse viral factors that modulate the host innate immune response (27–32). By comparing the SARS-CoV-2 genomic sequence to those of SARS-CoV and MERS-CoV, a number of viral proteins were predicted to exert similar functions to meddle in cellular innate signaling events (33). However, the actual viral proteins in SARS-CoV-2 and their mechanisms of action remain to be defined. Here, we identified the main protease of SARS-CoV-2, nonstructural protein 5 (Nsp5), as an inhibitor of the RIG-I–mitochondrial antiviral signaling (MAVS) protein–IFN pathway. Remarkably, Nsp5 demonstrates two distinct enzyme activities that differentially target RIG-I and MAVS. While Nsp5 proteolytically cleaves the 10 N-terminal amino acids from RIG-I, it functions as an E3 ligase to induce the ubiquitin- and proteosome-mediated degradation of MAVS. Nsp5-resistant mutants of RIG-I and MAVS restored the antiviral immune response and potently inhibited SARS-CoV-2 replication. A *de novo*-synthesized small-molecule inhibitor of Nsp5 restored the antiviral immune response and impeded SARS-CoV-2 protein processing, thus offering antiviral agents to combat the ongoing SARS-CoV-2 pandemic.

## RESULTS

### SARS-CoV-2 Nsp5 targets RIG-I for inhibition.

**FIG 1:**
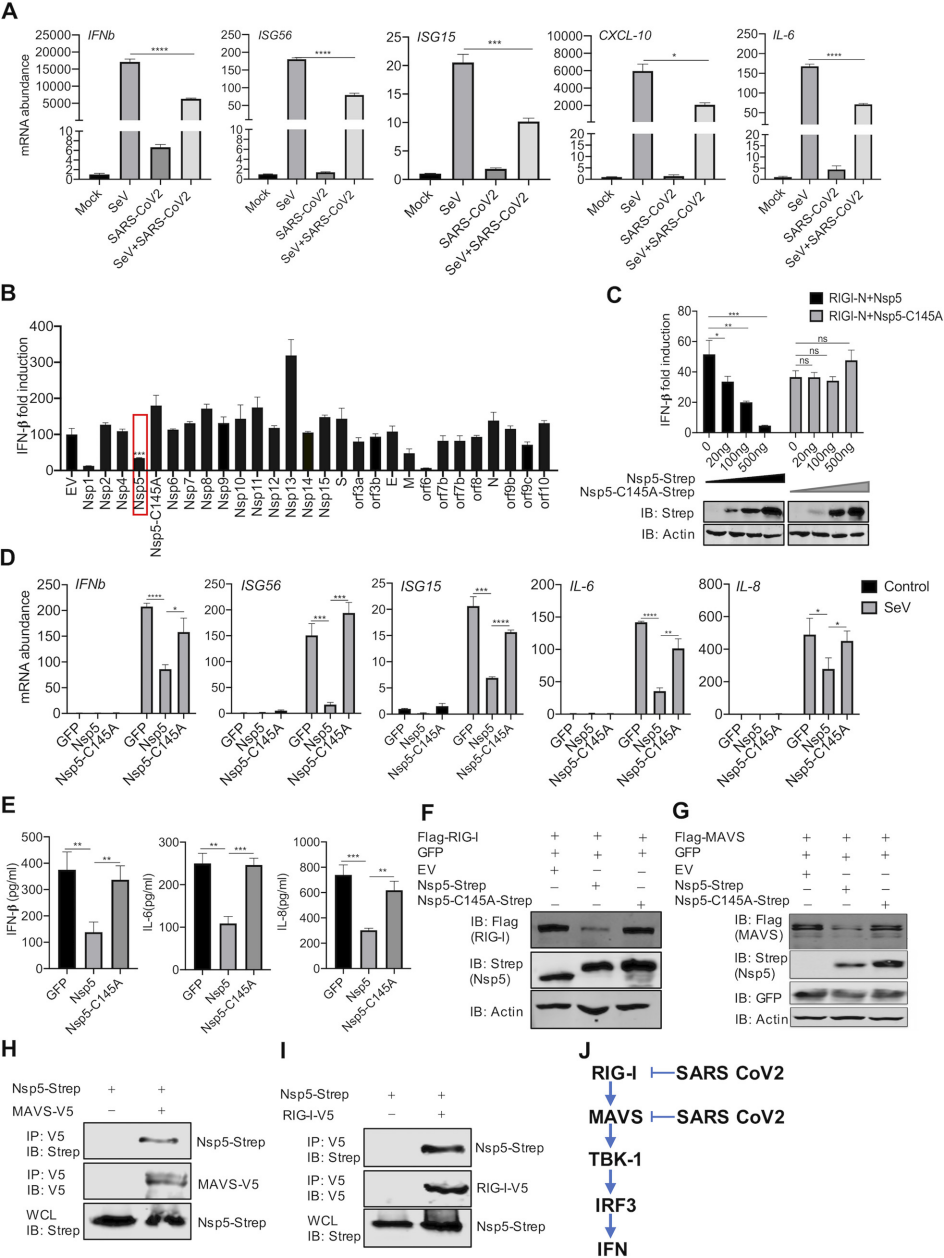
SARS-CoV-2 Nsp5 targets RIG-I and MAVS to inhibit IFN induction. (A) Calu-3 cells were mock infected or infected with SARS-CoV-2 (MOI = 0.5) for 24 h and superinfected with Sendai virus (SeV; 100 hemagglutinating units [HAU]/ml) for 6 h. The expression of the indicated antiviral genes was analyzed by real-time PCR using total RNA. (B) A screen identified SARS-CoV-2 proteins that inhibit IFN induction by the ectopically expressed MAVS in 293T cells. (C) IFN induction in 293T cells stimulated by RIG-I-N was assessed by a reporter assay with wild-type Nsp5 or its enzyme-deficient Nsp5-C145A mutant. IB, immunoblotting. (D, E) Antiviral cytokine gene expression in HCT116 cells stably expressing Nsp5 or the Nsp5-C145A mutant in response to SeV infection (100 HAU/ml) was analyzed by real-time PCR (D) and ELISA (E). (F, G) Immunoblotting analysis of ectopically expressed RIG-I (F) and MAVS (G) with Nsp5 or the Nsp5-C145A mutant. (H, I) Interactions of SARS-CoV-2 Nsp5 with RIG-I (H) or MAVS (I) were analyzed by coimmunoprecipitation in transfected 293T cells. (J) Diagram of the point of inhibition by SARS-CoV-2 of the RIG-I-MAVS pathway. Data are means ± standard deviations (SD). Significance was calculated using Student's two-tailed, unpaired *t* test. *, *P < *0.05; **, *P < *0.01; ***, *P < *0.001; ****, *P < *0.0001; ns, nonsignificant. See also Fig. S1 in the supplemental material.

SARS-CoV-2 is highly infectious and transmissible in the general human population (34). Ongoing studies have inferred that mechanisms beyond viral entry likely play an important role in this remarkably gained feature of SARS-CoV-2, compared with the transmissibility of SARS-CoV and MERS-CoV (35). We probed how SARS-CoV-2 interacts with the innate immune defense system using human Calu-3 lung cancer cells. When analyzed by real-time PCR, SARS-CoV-2 had minimal induction of type I interferons (IFNs) or IFN-stimulated genes, compared to that of Sendai virus (SeV) (Fig. 1A). Similar results were observed in normal human bronchial epithelial (NHBE) cells, with a much higher fold of induction (see Fig. S1A in the supplemental material). Interestingly, RNA derived from SARS-CoV-2-infected NHBE cells when transfected into NHBE cells induced IFN as potently as poly(I·C) (data not shown). These results suggest that SARS-CoV-2 actively blocks IFN induction to blunt the host antiviral immune response.

10.1128/mBio.02335-21.1 FIG S1 See also Fig. 1. (A) NHBE cells were mock infected or infected with SARS-CoV-2 (MOI = 0.5) for 24 h and superinfected with SeV (100 HAU/ml) for 6 h. The expression of the indicated antiviral genes was analyzed by real-time PCR using total RNA. (B, C) IFN gene expression in A549 stably expressing Nsp5 or the Nsp5-C145A mutant in response to Sendai virus infection (100 HAU/ml) was analyzed by real-time PCR (B) and ELISA (C). (D) IFN induction by a reporter assay using ectopic expression of RIG-I-N, MDA5-N, MAVS, TBK-1, and IRF3-5D was analyzed with Nsp5 or the Nsp5-C145A mutant in 293T cells. (E) Immunoblotting analysis of ectopically expressed MDA5, IKKβ, IKKε, TBK-1, and NEMO (IKKγ) in 293T cells with Nsp5 or the Nsp5-C145A mutant. Data are means ± SD. Significance was calculated using Student’s two-tailed, unpaired *t* test. ****, *P < *0.0001; ns, nonsignificant. Download FIG S1, JPG file, 0.3 MB. Copyright © 2021 Liu et al. 2021 Liu et al. https://creativecommons.org/licenses/by/4.0/ This content is distributed under the terms of the Creative Commons Attribution 4.0 International license.

To identify viral proteins that inhibit IFN induction, we screened a SARS-CoV-2 expression library with an IFN-β reporter assay induced by MAVS overexpression. MAVS is the adaptor molecule that relays innate immune activation downstream of RIG-I and MDA5. This assay identified several SARS-CoV-2 proteins, including Nsp1, Nsp5, and ORF6, that inhibit IFN induction to various extents (Fig. 1B). The inhibition of IFN induction by Nsp1 was due largely to the high basal induction of IFN by Nsp1 (data not shown), whereas ORF6 was reported to impede nuclear translocation of transcription factors such as IRF3 (36). Thus, we focused on Nsp5, the main protease of SARS-CoV-2, which is important for its replication. Importantly, the enzyme-deficient Nsp5-C145A mutant failed to inhibit IFN induction (Fig. 1B and C). These results show that Nsp5 inhibits IFN induction in an enzyme-dependent manner.

To characterize the Nsp5-mediated inhibition of the innate immune response, we profiled the expression of several inflammatory genes in response to Sendai virus infection. Real-time PCR analysis indicated that Nsp5, but not the enzyme-deficient Nsp5-C145A mutant, inhibited the expression of antiviral genes, including *IFNb*, *ISG56*, *ISG15*, *IL-6*, and *IL-8*, in HCT116 cells (Fig. 1D). The inhibition of gene expression also correlated with reduced cytokine production in the medium of Sendai virus-infected cells (Fig. 1E). Similar inhibition of inflammatory gene expression by Nsp5 was observed in Sendai virus-infected A549 cells (Fig. S1B and S1C).

The inhibition of the expression of both IRF3- and NF-κB-dependent genes by Nsp5 suggests that it targets upstream components of the RNA-sensing pathways. To determine the point of inhibition, we expressed key components of the RIG-I–IFN pathway, including constitutively active MDA5-N (2CARD), RIG-I-N (2CARD), IRF3-5D, MAVS, and TBK-1, and examined the IFN induction in the presence of Nsp5. As shown by an IFN reporter assay, we found that IFN induction was inhibited by Nsp5 when MDA5-N, RIG-I-N, and MAVS were overexpressed (Fig. S1D). However, IFN induced by the overexpression of TBK-1 and IRF3-5D was not affected by Nsp5. These results suggest that Nsp5 targets upstream molecules of the pathway, specifically MDA5, RIG-I, and/or MAVS. Indeed, transiently expressed RIG-I and MAVS proteins were reduced by Nsp5 as analyzed by immunoblotting (Fig. 1F and G), while other components of the innate immune pathway, including MDA5, IκB kinase β (IKKβ), IKKε, IKKγ (also known as NEMO), TBK-1, and TRAF6, were not affected by Nsp5 (Fig. S1E). Thus, the inhibition of MDA5-mediated IFN induction by Nsp5 is likely due to its effect on MAVS. We also investigated Nsp5 interaction with RIG-I or MAVS by coimmunoprecipitation (Co-IP). As shown in Fig. 1H and I, both RIG-I and MAVS precipitated with Nsp5, supporting their interactions. These results collectively show that SARS-CoV-2 Nsp5 targets RIG-I and MAVS to inhibit the antiviral immune response (Fig. 1J).

### Nsp5 cleaves RIG-I at the Q10 residue.

**FIG 2:**
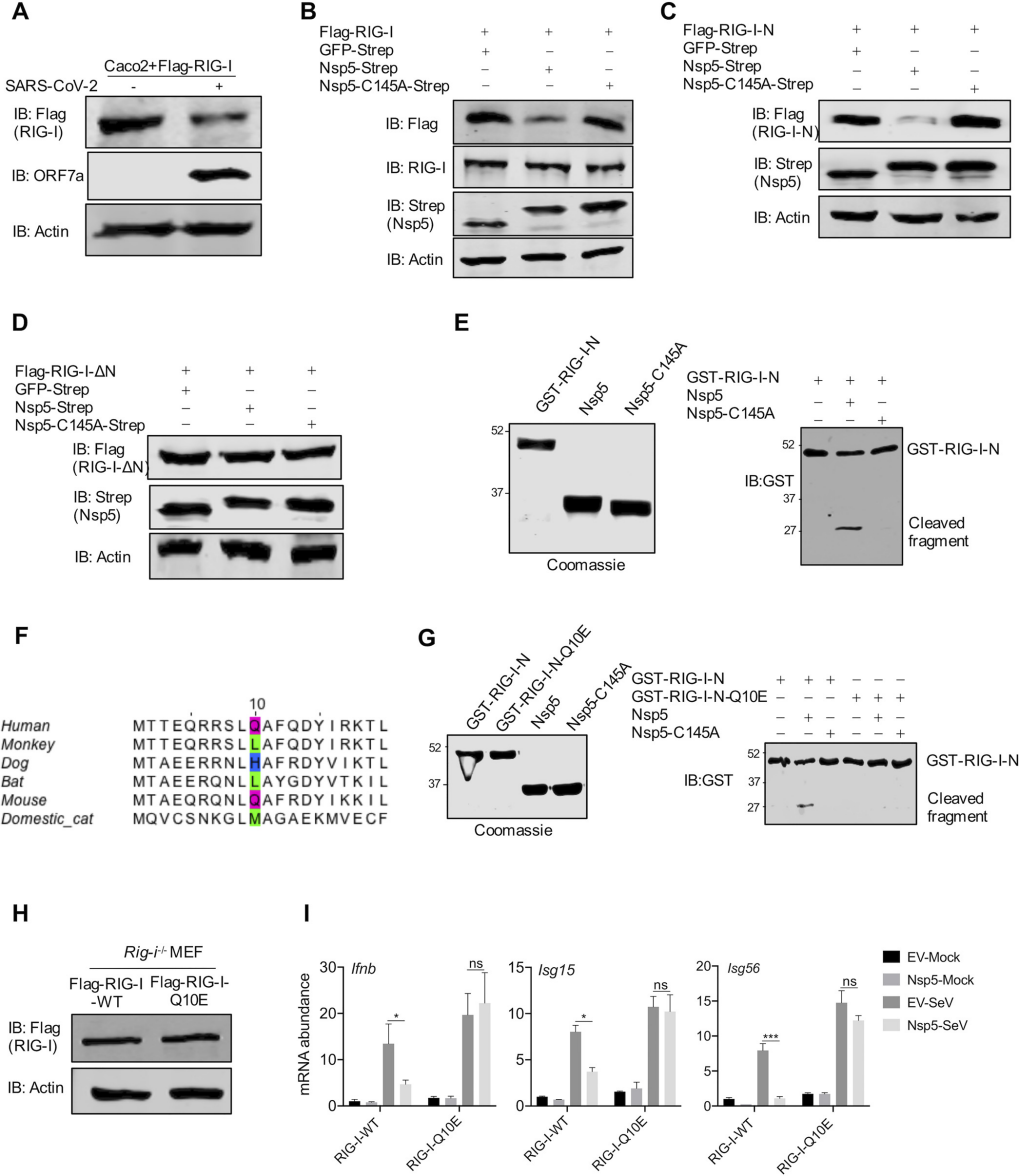
Nsp5 cleaves RIG-I at the Q10 residue. (A, B) Immunoblotting analysis of ectopically expressed RIG-I in Caco-2 cells infected with SARS-CoV-2 (A) and with the expression of Nsp5 or the Nsp5-C145A mutant using anti-Flag antibody or anti-RIG-I antibody (B). (C, D) Immunoblotting analysis of ectopically expressed RIG-I-N (C) and RIG-I-ΔN (D) in 293T cells with the expression of Nsp5 or the Nsp5-C145A mutant. (E, right) Numbers at the left of the gel are molecular weights in kDa. (E) Immunoblotting analysis of *in vitro* GST–RIG-I-N cleavage by Nsp5 or the Nsp5-C145A mutant with all proteins purified from E. coli and analyzed using Coomassie blue staining (left). (F) Alignment of the 20 N-terminal amino acids of RIG-I from human and five nonhuman mammalian species. The putative cleavage site, Q10, of human RIG-I and its equivalent residues are highlighted with colored boxes. (G) Immunoblotting analysis of GST–RIG-I-N and GST–RIG-I-N-Q10E after *in vitro* cleavage by Nsp5 or the Nsp5-C145A mutant with all proteins purified from E. coli and analyzed using Coomassie blue staining (left). (H) Immunoblotting analysis of whole-cell lysates of *Rig-i^−/−^* MEFs reconstituted with RIG-I-WT or RIG-I-Q10E. (I) Total RNA extracted from these cells without or with Nsp5 expression in response to Sendai virus infection (100 HAU/ml) was analyzed by RT-qPCR with primers specific for the indicated genes. Data are means ± SD. Significance was calculated using Student’s two-tailed, unpaired *t* test. *, *P < *0.05; ***, *P < *0.001; ns, nonsignificant. See also Fig. S2.

To probe the biochemical basis of RIG-I inhibition by Nsp5, we analyzed RIG-I by immunoblotting. We first examined RIG-I downregulation by SARS-CoV-2 using the Caco-2 cell line, which stably expresses RIG-I with a Flag epitope at the N terminus. As shown by immunoblotting analysis, we found that SARS-CoV-2 infection and Nsp5 expression decreased Flag–RIG-I protein in Caco-2 cells and 293T cells (Fig. 2A and B). Surprisingly, no difference in RIG-I protein was observed in immunoblots analyzed with anti-RIG-I antibody (Fig. 2B). These results suggest that Nsp5 removes a very small N-terminal fragment, such that the apparent molecular weight of the main RIG-I fragment did not significantly change. To test this idea, we used the Flag–RIG-I-N and Flag–RIG-I-ΔN (deletion of 2CARD) constructs. Indeed, Nsp5 reduced the protein size of Flag–RIG-I-N but not that of Flag–RIG-I-ΔN (Fig. 2C and D). To determine whether Nsp5 can function as a bona fide protease for RIG-I, we purified glutathione *S*-transferase (GST)–RIG-I-N, Nsp5, and the enzyme-deficient Nsp5-C145A mutant from bacteria and performed an *in vitro* cleavage assay. As shown in Fig. 2E, Nsp5, but not Nsp5-C145A, produced a fragment of approximately the size of GST (27 kDa). This result supports the conclusion that Nsp5 cleaves the very N-terminal end of RIG-I-N, without altering the apparent size of GST.

10.1128/mBio.02335-21.2 FIG S2 See also Fig. 2. (A, B) Immunoblotting analysis of ectopically expressed Flag-RIG-I-WT and Flag-RIG-I-Q10E (A) or RIG-I-WT-V5 and RIG-I-Q10E-V5 (B) with wild-type Nsp5 or the Nsp5-C145A mutant using whole-cell lysates of transfected 293T cells, with the indicated antibodies. Download FIG S2, JPG file, 0.1 MB. Copyright © 2021 Liu et al. 2021 Liu et al. https://creativecommons.org/licenses/by/4.0/ This content is distributed under the terms of the Creative Commons Attribution 4.0 International license.

To identify the site of cleavage within the N terminus of RIG-I, we aligned the 20 N-terminal amino acids of RIG-I proteins of humans and several mammals, including monkey, dog, bat, mouse, and cat. The alignment showed that a putative LQA Nsp5 cleavage sequence is shared only by human and mouse RIG-I proteins (Fig. 2F). Importantly, L and A flanking the putative Q cleavage residue favor the cleavage by Nsp5 as previous studies showed (37–39). Interestingly, the preferred cleavage site was not shared by RIG-I proteins of bat, cat, dog, and monkey, suggesting that cells of these mammals are likely more resistant to SARS-CoV-2 replication. To determine whether Nsp5 targets Q10 of human RIG-I, we utilized a RIG-I mutant, RIG-I-Q10E, which is the RIG-I deamidated by murine gamma herpesvirus 68 (MHV68) (40). Remarkably, RIG-I-Q10E was resistant to Nsp5-induced downregulation, while wild-type RIG-I (RIG-I-WT) was not (Fig. S2A). In contrast, when RIG-I and RIG-I-Q10E constructs with a V5 epitope at their carboxyl terminus were expressed in the presence of Nsp5, RIG-I and RIG-I-Q10E demonstrated no difference in their apparent molecular weights (Fig. S2B). We also performed an *in vitro* cleavage assay using GST–RIG-I-N, GST–RIG-I-N-Q10E, Nsp5, and the enzyme-deficient Nsp5-C145A proteins purified from bacteria. As shown in Fig. 2G, GST–RIG-I-N, but not GST–RIG-I-N-Q10E, produced a fragment of approximately the size of GST in the presence of Nsp5. These results imply that there is no additional cleavage site for Nsp5. Thus, Nsp5 targets the Q10 residue of human RIG-I for cleavage.

To probe the biological consequence of Nsp5-mediated cleavage of RIG-I, we reconstituted *Rig-i^−/−^* mouse embryonic fibroblasts (MEFs) with wild-type RIG-I and RIG-I-Q10E (Fig. 2H). When Nsp5 MEFs were infected with Sendai virus, we found that the expression of antiviral genes, including *Ifnb*, *Isg15*, and *Isg56*, was greatly reduced by Nsp5 in *Rig-i^−/−^* MEFs reconstituted with wild-type RIG-I (Fig. 2I). In contrast, Nsp5 had no apparent effect on the antiviral gene expression in *Rig-i^−/−^* MEFs reconstituted with RIG-I-Q10E. These results support the conclusion that RIG-I-Q10E resists Nsp5-mediated cleavage and immune evasion.

### Loss-of-function and dominant negative effect of the cleaved RIG-I fragment.

**FIG 3:**
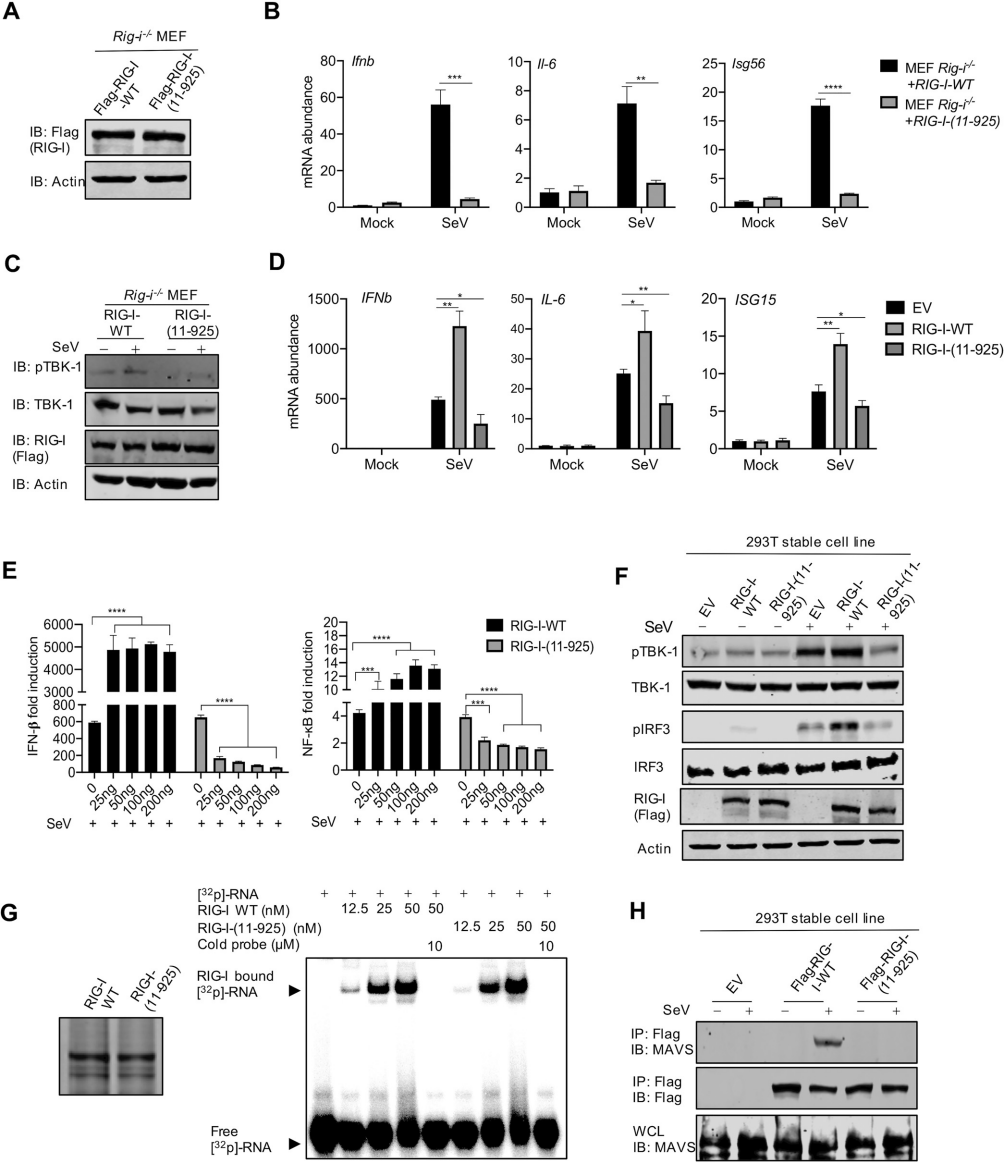
Loss of function and dominant negative effect of the cleaved RIG-I fragment [RIG-I-(11–925)]. (A) Immunoblotting analysis of WCLs of *Rig-i^−/−^* MEFs reconstituted with RIG-I-WT or RIG-I-Q10E. (B) Total RNA extracted from *Rig-i^−/−^* MEFs reconstituted with RIG-I-WT or RIG-I-11-925 infected with Sendai virus (100 HAU/ml) was analyzed by RT-qPCR with primers specific for the indicated genes. (C) Immunoblotting analysis of WCLs of *Rig-i^−/−^* MEFs reconstituted with RIG-I-WT or RIG-I-(11–925) and infected with Sendai virus (100 HAU/ml) with the indicated antibodies. (D) Total RNA extracted from 293T cells stably expressing RIG-I-WT or RIG-I-(11–925) infected with Sendai virus (100 HAU/ml) was analyzed by RT-qPCR with primers specific for the indicated genes. (E) IFN induction and NF-κB activation in 293T cells transfected with increasing amounts of plasmids containing RIG-I or RIG-I-(11–925) were analyzed by luciferase reporter assay. (F) Immunoblotting analysis of WCLs of 293T cells stably expressing RIG-I-WT or RIG-I-11-925 with the indicated antibodies. (G) Electrophoresis mobility shift assay of purified RIG-I or RIG-I-(11–925) incubated with ^32^P-labeled 5′-triphosphate 19-mer dsRNA. (H) Immunoblotting analysis of precipitated RIG-I (anti-Flag) and WCLs of 293T cells that stably express RIG-I-WT or RIG-I-(11–925) and that were infected with Sendai virus (100 HAU/ml) with the indicated antibodies. Data are means ± SD. Significance was calculated using Student’s two-tailed, unpaired *t* test. *, *P < *0.05; **, *P < *0.01; ***, *P < *0.001; ****, *P < *0.0001. See also Fig. S3.

Previously, we reported that MHV68 targets the Q10 residue of RIG-I for deamidation to evade cytokine production (40). The fact that SARS-CoV-2 Nsp5 also targets the same residue for cleavage suggests that the 10 N-terminal amino acids are critical for RIG-I function. Indeed, a previous study reported that the loss of the 10 N-terminal amino acids impaired the ability of RIG-I-N to associate with free ubiquitin chains and to activate IRF3 *in vitro* (41). Thus, we characterized the function of RIG-I-(11–925) in activating the innate immune defense. First, we reconstituted *Rig-i^−/−^* MEFs with wild-type RIG-I and RIG-I-(11–925) via lentiviral infection (Fig. 3A). When antiviral gene expression was examined, we found that Sendai virus infection potently induced the expression of *Ifnb*, *Isg56*, and *Il-6* in *Rig-i^−/−^* MEFs reconstituted with wild-type RIG-I but not in those reconstituted with RIG-I-(11–925) (Fig. 3B). This result was also consistent with the phosphorylation and activation of TBK-1 in *Rig-i^−/−^* MEFs reconstituted with wild-type RIG-I, which was not seen in those reconstituted with RIG-I-(11–925) (Fig. 3C). To determine whether RIG-I-(11–925) has a dominant negative effect, we performed reporter assays for IFN induction and NF-κB activation upon Sendai virus infection. While overexpression of wild-type RIG-I elevated IFN induction and NF-κB activation, overexpression of RIG-I-(11–925) potently reduced IFN induction and NF-κB activation in a dose-dependent manner (Fig. 3E). Similarly, real-time PCR analysis further showed that RIG-I-(11–925) inhibited the expression of inflammatory genes, including *IFNb* and *IL-6* (Fig. S3A). Consistently with reduced inflammatory gene expression, RIG-I-(11–925) also inhibited the phosphorylation of IRF3 and TBK-1, signaling events downstream of RIG-I and MAVS, in response to Sendai virus infection (Fig. 3F). Thus, RIG-I-(11–925) inhibits innate immune activation induced by dsRNA.

10.1128/mBio.02335-21.3 FIG S3 See also Fig. 3. (A) RT-qPCR was performed with total RNA extracted from 293T cells that were transfected with plasmids containing RIG-I-WT or RIG-I-11-925 and infected with Sendai virus (100 HAU/ml). (B) Immunoblotting analysis of precipitated RIG-I (anti-Flag) and whole-cell lysates of 293T cells stably expressing RIG-I-WT, which were transfected with increasing amounts of a plasmid containing RIG-I-(11–925), and infected with Sendai virus (100 HAU/ml) with the indicated antibodies. Data are means ± SD. Significance was calculated using Student’s two-tailed, unpaired *t* test. **, *P < *0.01; ***, *P < *0.001; ****, *P < *0.0001; ns, nonsignificant. Download FIG S3, JPG file, 0.2 MB. Copyright © 2021 Liu et al. 2021 Liu et al. https://creativecommons.org/licenses/by/4.0/ This content is distributed under the terms of the Creative Commons Attribution 4.0 International license.

To dissect the mechanism of inhibition, we determined whether RIG-I-(11–925) can bind dsRNA as we previously described (40). Indeed, an RNA gel shift assay showed that RIG-I-(11–925), like wild-type RIG-I, retarded dsRNA migration in a dose-dependent manner (Fig. 3G). This result indicates that RIG-I-(11–925) is capable of binding dsRNA, consistent with the notion that the carboxyl-terminal domain of RIG-I is sufficient for dsRNA binding. Finally, we assessed the effect of RIG-I-(11–925) on the heterodimerization between RIG-I and MAVS, induced by Sendai virus infection. Coimmunoprecipitation assay indicated that wild-type RIG-I, but not RIG-I-(11–925), pulled down MAVS in Sendai virus-infected cells (Fig. 3H). Moreover, the overexpression of RIG-I-(11–925) inhibited RIG-I-MAVS heterodimerization in a dose-dependent manner in response to Sendai virus infection (Fig. S3B). Taken together, these results collectively demonstrate that RIG-I-(11–925) exerts a dominant negative effect on dsRNA-induced innate immune activation, likely via competing with RIG-I for binding to dsRNA.

### Nsp5 promotes MAVS degradation via the ubiquitin-proteosome pathway.

**FIG 4:**
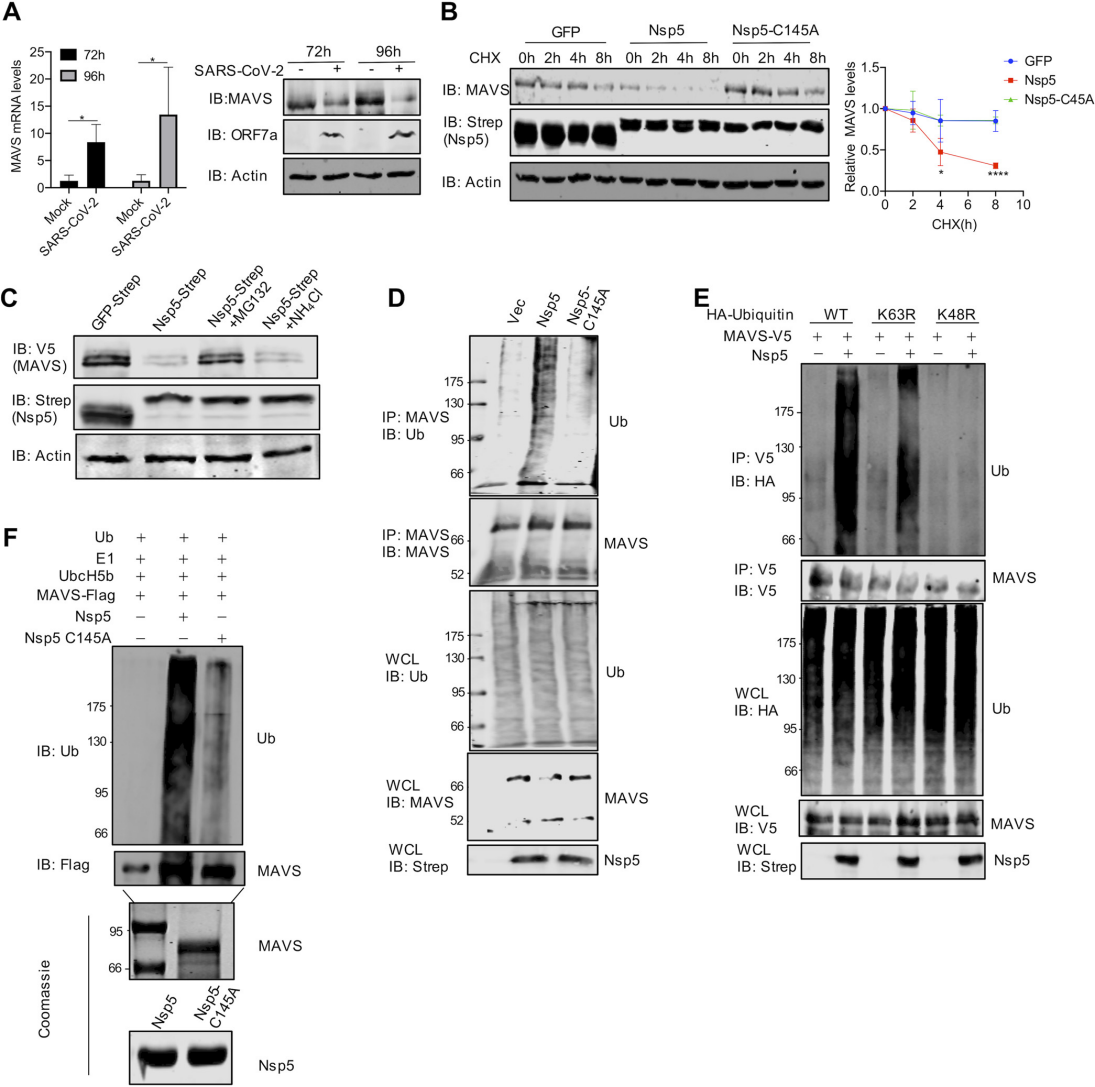
Nsp5 promotes MAVS ubiquitination and degradation. (A) qRT-PCR analysis and immunoblotting analysis of Caco-2 cells infected with SARS-CoV-2 (MOI = 1) for 72 h and 96 h. (B) Immunoblotting analysis of WCLs of 293T cells transfected with plasmids containing Nsp5 or Nsp5C145A and treated with DMSO or 100 μg/ml cycloheximide (CHX) and with the indicated antibodies. MAVS was quantified by densitometry and plotted as shown on the right. Results represent one of three independent experiments. (C) Immunoblotting analysis of WCLs of 293T cells transfected with plasmids containing MAVS and Nsp5 and treated with the proteasome inhibitor MG132 (10 μM) or the lysosome inhibitor NH_4_Cl (10 mM). (D) Immunoblotting analysis of endogenous ubiquitination levels of MAVS and WCLs of 293T cells transfected with plasmids containing Nsp5 or Nsp5-C145A and treated with the indicated antibodies. Vec, vector; Ub, ubiquitin. (E) Immunoblotting analysis of endogenous ubiquitination levels of MAVS and WCLs of 293T cells transfected with plasmids containing Nsp5 or Nsp5-C145A and ubiquitin (WT) and treated with the K48R or K63R mutant and with the indicated antibodies. (F) *In vitro* ubiquitination assay of MAVS. Nsp5 or Nsp5-C145A and MAVS were incubated in a reaction mixture containing ubiquitin, E1, and E2(UbcH5b), and ubiquitination levels were determined by immunoblotting with anti-Ub antibody. Purified MAVS is shown at the bottom with SDS-PAGE and Coomassie blue staining. Data are means ± SD. Significance was calculated using Student's two-tailed, unpaired *t* test. *, *P < *0.05.

MAVS is a mitochondrion-resident protein that relays innate immune activation downstream of RIG-I and MDA5 (20–23). To validate the supposition that Nsp5 reduces MAVS protein during SARS-CoV-2 infection, we examined MAVS expression in SARS-CoV-2-infected Caco-2 cells by immunoblotting and quantitative real-time PCR (qRT-PCR). Interestingly, SARS-CoV-2 infection increased MAVS mRNA, but greatly diminished MAVS protein, at 72 and 96 h postinfection (Fig. 4A). To determine whether Nsp5 targets MAVS for proteolytic cleavage, we inspected the primary amino acid sequence of MAVS for possible consensus Nsp5 cleavage sites. Although several putative sites with low confidence were identified, mutations that change these putative cleavage glutamines to glutamates did not restore MAVS protein in the presence of Nsp5 (data not shown). Additionally, we did not detect any cleaved MAVS fragments by immunoblotting (data not shown). These results suggest that Nsp5 may reduce MAVS protein independently of its protease activity. To test this hypothesis, we measured the half-life of MAVS when translation was inhibited with cycloheximide. As shown in Fig. 4B, Nsp5 reduced the half-life of MAVS to approximately 4 h, whereas its half-life in the presence of Nsp5-C145A or green fluorescent protein (GFP) was calculated to be >8 h. Thus, Nsp5 increases MAVS degradation to reduce MAVS protein expression. In mammalian cells, there are two main protein-degradative pathways, i.e., the proteosome and lysosome. To determine which of these pathways is required for Nsp5-mediated MAVS degradation, we treated cells with MG132, a pan-proteosome inhibitor, and NH_4_Cl, a lysosome inhibitor. Remarkably, MG132 greatly restored MAVS protein in the presence of Nsp5, whereas NH_4_Cl had no apparent effect on MAVS protein (Fig. 4C). These results support the conclusion that Nsp5 induces MAVS degradation via proteosomes.

Proteosomes remove proteins that are tagged with ubiquitin via proteolytic cleavage. Thus, we first determined whether MAVS undergoes ubiquitination. When endogenous MAVS was precipitated and analyzed with antiubiquitin antibody, we found that Nsp5 potently increased the ubiquitination of MAVS but that Nsp5-C145A failed to do so (Fig. 4D). Ubiquitin can be added to target proteins, such as MAVS, via distinct lysine residues of ubiquitin. Two main lysine residues, K48 and K63, are best characterized for their distinct functions in mammalian cells. In general, K63-linked ubiquitin chains mark proteins for signal transduction, while K48-linked ubiquitin chains tag proteins for proteosome-mediated degradation. To determine the role of K63 and K48 of ubiquitin in MAVS degradation, we overexpressed K63R and K48R ubiquitin mutants and examined MAVS ubiquitination. This assay showed that the ubiquitin-K48R mutant, but not the ubiquitin-K63R mutant, reduced MAVS ubiquitination to background levels (Fig. 4E), supporting the conclusion that Nsp5 promotes the K48-linked ubiquitination of MAVS. We next determined whether Nsp5 can function as a bona fide E3 ligase, given that Nsp5 is characterized as a cysteine protease. When Nsp5 was purified from bacteria and added to MAVS along with E1 and E2(UbcH5b), a massive ubiquitin signal of higher molecular weight (>72 kDa) was detected in the presence of Nsp5 by immunoblotting (Fig. 4F). In contrast, residual levels of ubiquitin signal were detected in the presence of Nsp5-C145A. Collectively, these results demonstrate that Nsp5 can function as a bona fide E3 ligase to promote MAVS degradation via the ubiquitin-proteosome system.

### Nsp5 targets the K136 residue of MAVS to promote protein degradation.

**FIG 5:**
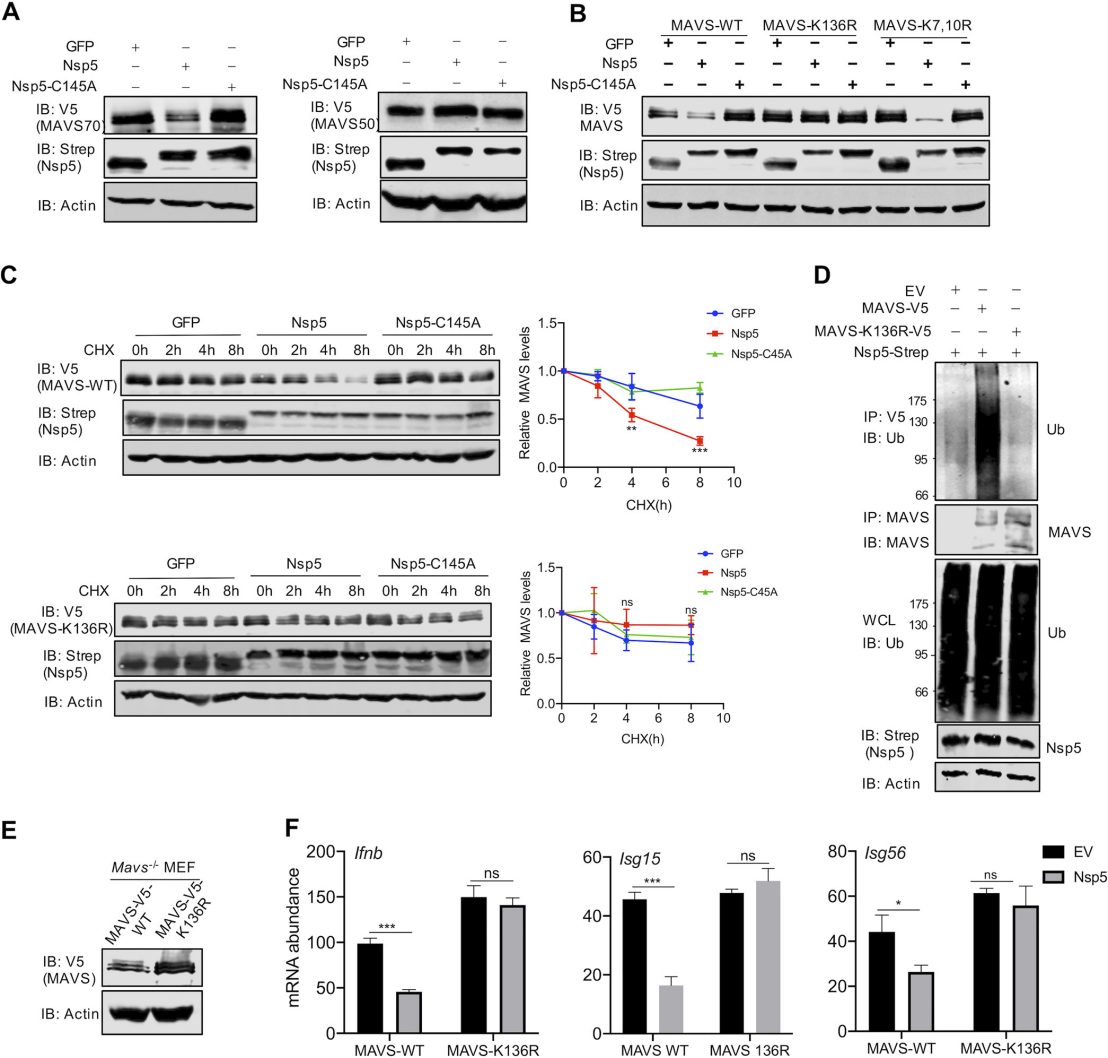
Nsp5 targets the K136 residue of MAVS to promote ubiquitination and degradation. (A) Immunoblotting analysis of WCLs of 293T cells transfected with plasmids containing MAVS70 or MAVS50, together with those containing Nsp5 or Nsp5-C145A, and treated with the indicated antibodies. (B) Immunoblotting analysis of WCLs of 293T cells transfected with plasmids containing wild-type MAVS, MAVS-K136R, or MAVS-K7R/K10R and those containing Nsp5 or Nsp5-C145A and treated with the indicated antibodies. (C) Immunoblotting analysis of WCLs of 293T cells stably expressing MAVS-WT or MAVS-K136R transfected with plasmids containing Nsp5 or Nsp5C145A, treated with DMSO or cycloheximide (CHX, 100 μg/ml), and treated with the indicated antibodies. MAVS protein was quantified by densitometry and plotted as shown on the right. (D) Immunoblotting analysis of ubiquitination levels of MAVS and WCLs of 293T cells stably expressing MAVS-WT or MAVS-K136R transfected with plasmids containing Nsp5 and treated with the indicated antibodies. (E) Immunoblotting analysis of WCLs of *Mavs^−/−^* MEFs reconstituted with MAVS-WT or MAVS-K136R. (F) Total RNA extracted from these cells without or with Nsp5 expression was analyzed by RT-qPCR and treated with primers specific for the indicated genes. See also Fig. S4.

Having demonstrated that Nsp5 promotes MAVS ubiquitination, we searched for the ubiquitination site(s) within MAVS. One interesting feature of MAVS is that there are multiple forms of MAVS expressed from the same transcript via internal translation initiation, giving rising to three isoforms of ∼70, 50, and 30 kDa, thus referred to as MAVS70, MAVS50, and MAVS30, respectively (42–44). We explored these isoforms as natural truncations from the N terminus and examined the effect of Nsp5 on their expression. Remarkably, while Nsp5 greatly reduced the expression of the MAVS70 protein, it had no apparent effect on MAVS50, which lacks the first 140 amino acids of MAVS70, compared to the effects of the Nsp5-C145A mutant (Fig. 5A). This result suggests that the N-terminal sequence (amino acids 1 to 140) is important for Nsp5-mediated downregulation of MAVS70. Inspection of the 140 N-terminal amino acids identified three lysine residues, K7, K10, and K136, that can potentially function as ubiquitination receptors. We generated two MAVS mutants containing either a K7, K10R, or K136R mutation. In transfected 293T cells, the MAVS-K7,10R mutant and, to a lesser extent, MAVS-K136R were expressed at higher levels than wild-type MAVS (Fig. 5B). Nsp5 expression detectably reduced the protein levels of MAVS-K7,10R and that of wild-type MAVS. However, Nsp5 had a marginal effect on the protein level of MAVS-K136R, which was comparable to that of Nsp5-C145A. These results suggest that Nsp5 targets the K136 residue of MAVS for ubiquitination and degradation.

10.1128/mBio.02335-21.4 FIG S4 See also Fig. 5. (A) Immunoblotting analysis of whole-cell lysates of 293T cells stably expressing MAVS-TM transfected with plasmids containing Nsp5 or Nsp5C145A and treated with DMSO or 100 μg/ml cycloheximide (CHX), with the indicated antibodies. MAVS protein was quantified by densitometry and plotted as shown on the right. (B) IFN induction by a reporter assay using ectopic expression of MAVS-WT or MAVS-K136R was analyzed with Nsp5 or the Nsp5-C145A mutant in 293T cells. Download FIG S4, JPG file, 0.1 MB. Copyright © 2021 Liu et al. 2021 Liu et al. https://creativecommons.org/licenses/by/4.0/ This content is distributed under the terms of the Creative Commons Attribution 4.0 International license.

To further validate that Nsp5 targets the K136 residue of MAVS for degradation, we determined the half-life of wild-type MAVS and MAVS-K136R in the presence of GFP, Nsp5, and Nsp5-C145A. As with endogenous MAVS, exogenously expressed MAVS had a half-life of more than 8 h. However, Nsp5 reduced MAVS’s half-life to approximately 4 h (Fig. 5C). In stark contrast, MAVS-K136R demonstrated a half-life of >8 h in the presence of GFP, Nsp5, or Nsp5-C145A. The MAVS mutant containing all three K→R mutations (triple mutant [TM]) behaved similarly to MAVS-K136R in its half-life (Fig. S5A). When MAVS ubiquitination was examined in stable 293T cells, we found that MAVS-K136R had a background level of ubiquitin signal, while ubiquitination of wild-type MAVS was readily detected under similar conditions (Fig. 5D). These results collectively show that the K136 residue is targeted by Nsp5 for ubiquitination to promote MAVS degradation.

**FIG 6:**
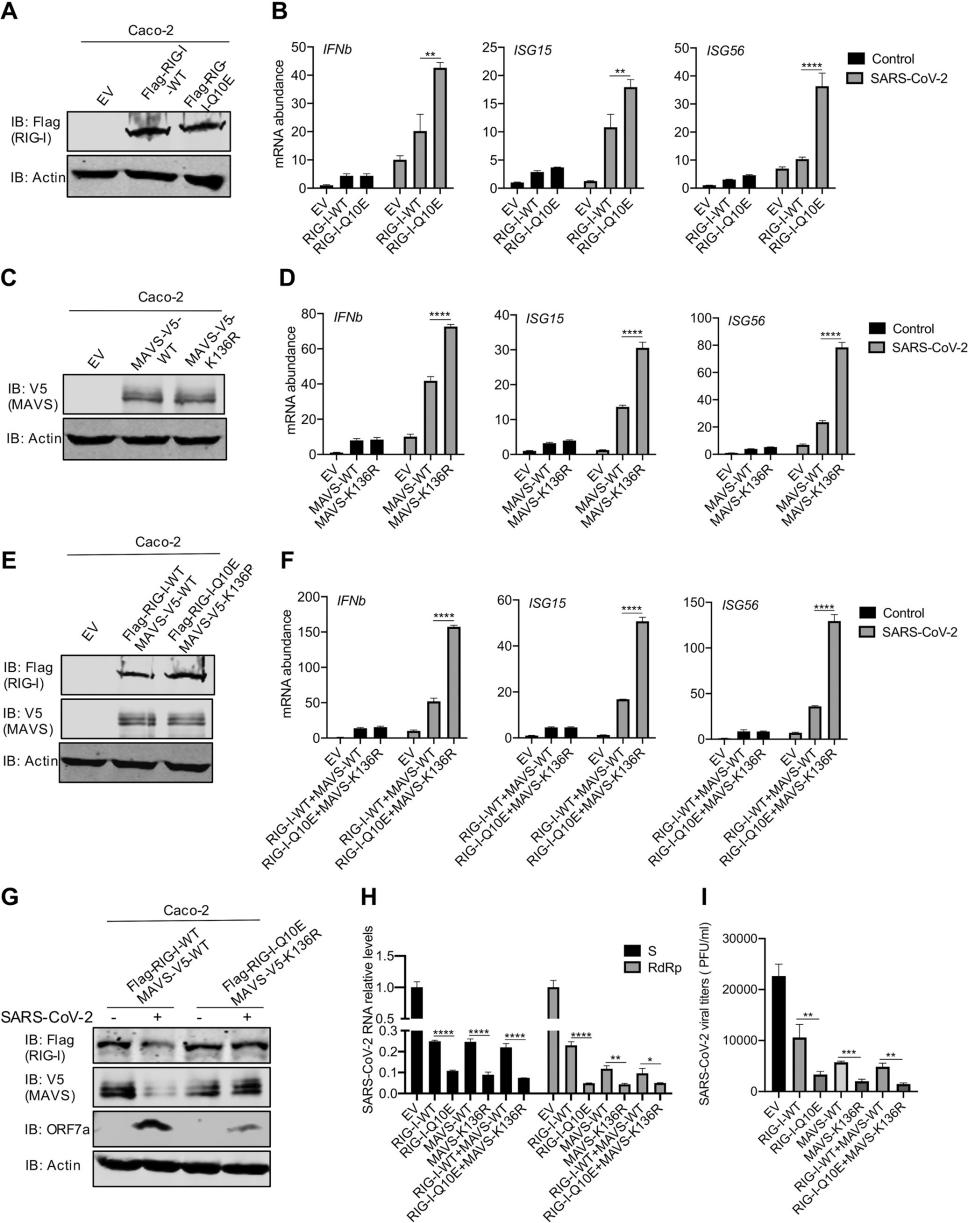
RIG-I-Q10E and MAVS-K136A resist destruction and restore innate immune activation during SARS-CoV-2 infection. (A, B) Immunoblotting analysis of WCLs of Caco-2 cells stably expressing RIG-I-WT or RIG-I-Q10E with the indicated antibodies (A). Total RNA extracted from Caco-2 cells stably expressing RIG-I-WT or RIG-I-Q10E infected with SARS-CoV-2 (MOI = 1) was analyzed by RT-qPCR with primers specific for the indicated genes (B). (C) Immunoblotting analysis of WCLs of Caco-2 cells stably expressing MAVS-WT or MAVS-K136R with the indicated antibodies. (D) Total RNA extracted from Caco-2 cells stably expressing MAVS-WT or MAVS-K136R infected with SARS-CoV-2 (MOI = 1) was analyzed by RT-qPCR with primers specific for the indicated genes. (E) Immunoblotting analysis of WCLs of Caco-2 cells stably expressing RIG-I-WT and MAVS-WT or RIG-I-Q10E and MAVS-K136R with the indicated antibodies. (F) Total RNA extracted from Caco-2 cells stably expressing RIG-I-WT and RIG-I-Q10E or MAVS-WT and MAVS-K136R infected with SARS-CoV-2 (MOI = 1) was analyzed by RT-qPCR with primers specific for the indicated genes. (G) Immunoblotting analysis of WCLs of Caco-2 cells stably expressing RIG-I-WT and RIG-I-Q10E or MAVS-WT and MAVS-K136R infected with SARS-CoV-2 for 72 h, with the indicated antibodies. (H, I) SARS-CoV-2 replication in Caco-2 cells stably expressing RIG-I-WT, RIG-I-Q10E, MAVS-WT, MAVS-K136R, RIG-I-WT, and MAVS-WT or RIG-I-Q10E and MAVS-K136R was analyzed by RT-qPCR for SARS-CoV-2 S and RdRp RNA (H) or determined by plaque assay (I) at 72 h postinfection. Data are means ± SD. Significance was calculated using Student’s two-tailed, unpaired *t* test. *, *P < *0.05; **, *P < *0.01; ***, *P < *0.001; ****, *P < *0.0001. See also Fig. S5.

10.1128/mBio.02335-21.5 FIG S5 See also Fig. 6. (A, B) *Rig-i*^−/−^ MEFs stably expressing hACE2 were reconstituted with RIG-I-WT or RIG-I-Q10E and infected with SARS-CoV-2 (MOI = 0.1) for 24 h. (A) Whole-cell lysates were analyzed with the indicated antibodies by immunoblotting. (B) Total RNA was extracted for reverse transcription. IFN-β activation was analyzed by real-time PCR. (C to F) *Mavs*^−/−^ MEFs stably expressing hACE2 were reconstituted with MAVS-WT or MAVS-K136R, RIG-I-WT, and MAVS-WT, or RIG-I-Q10E and MAVS-K136R, and cells were infected with SARS-CoV-2 (MOI = 0.1) for 24 h. (C and E) Whole-cell lysates were analyzed with the indicated antibodies by immunoblotting. (D and F) Total RNA was extracted for reverse transcription. IFN-β activation was analyzed by real-time PCR. (G) SARS-CoV-2 replication in *Rig-i*^−/−^ MEFs stably expressing hACE2 reconstituted with EV, RIG-I-WT, or RIG-I-Q10E, MAVS-WT, MAVS-K136R, RIG-I-WT, and MAVS-WT, or RIG-I-Q10E and MAVS-K136R was analyzed by RT-qPCR for SARS-CoV-2 RNA at 24 h postinfection. (H, I) SARS-CoV-2 replication in *Mavs*^−/−^ MEFs stably expressing hACE2 reconstituted with EV, MAVS-WT, MAVS-K136R (H), RIG-I-WT and MAVS-WT, or RIG-I-Q10E and MAVS-K136R (I) was analyzed by RT-qPCR for SARS-CoV-2 RNA at 24 h postinfection. Data are means ± SD. Significance was calculated using Student’s two-tailed, unpaired *t* test. ***P < 0.001; ****, *P < *0.0001; ns, nonsignificant. Download FIG S5, JPG file, 0.3 MB. Copyright © 2021 Liu et al. 2021 Liu et al. https://creativecommons.org/licenses/by/4.0/ This content is distributed under the terms of the Creative Commons Attribution 4.0 International license.

To assess the biological consequence of Nsp5-mediated degradation of MAVS, we reconstituted *Mavs^−/−^* mouse embryonic fibroblasts (MEFs) with wild-type MAVS and MAVS-K136R (Fig. 5E). We found that the expression of antiviral genes, including *Ifnb*, *Isg15*, and *Isg56*, was greatly reduced by Nsp5 in *Mavs^−/−^* MEFs reconstituted with wild-type MAVS (Fig. 5F). In contrast, Nsp5 had no apparent effect on the antiviral gene expression in *Mavs^−/−^* MEFs reconstituted with MAVS-K136R. Similar results were observed in an IFN reporter assay; IFN induction by wild-type MAVS, but not that by MAVS-K136R, was inhibited by Nsp5 (Fig. S4B). These results support the conclusion that MAVS-K136R resists Nsp5-mediated degradation and immune evasion.

### RIG-I-Q10E and MAVS-K136R resist destruction and restore innate immune activation during SARS-CoV-2 infection.

Considering that SARS-CoV-2 deploys Nsp5 to simultaneously inactivate RIG-I and MAVS, we reasoned that endogenous RIG-I and MAVS are functionally nullified by SARS-CoV-2 during infection. Thus, we ectopically expressed wild-type RIG-I or RIG-I-Q10E in human Caco-2 cells (Fig. 6A) and examined the expression of antiviral genes, including *IFNb*, *ISG15*, and *ISG56*. Upon SARS-CoV-2 infection, we observed clear yet weak induction of these genes in control (EV) Caco-2 cells (Fig. 6B). Ectopic expression of wild-type RIG-I further elevated the expression of these genes by 1-fold for *IFNb* and *ISG56* and by 10-fold for *ISG15*. Compared to wild-type RIG-I, RIG-I-Q10E further elevated the expression of these antiviral genes by 1-fold. These results were reproduced from *Rig-i*^−/−^ MEFs expressing wild-type RIG-I and RIG-I-Q10E (Fig. S5A and S5B). Very similar results were observed for the expression of wild-type MAVS and MAVS-K136R in both Caco-2 cells (Fig. 6C and D) and *Mavs^−/−^* MEFs (Fig. S5C and S5D) in response to SARS-CoV-2 infection. Next, we generated Caco-2 cells that express either wild-type RIG-I plus wild-type MAVS or RIG-I-Q10E plus MAVS-K136R and infected them with SARS-CoV-2 (Fig. 6E). Upon SARS-CoV-2 infection, we observed clear reduction in ectopically expressed wild-type RIG-I and MAVS proteins (Fig. 6G). In contrast, RIG-I-Q10E and MAVS-K136R were not significantly reduced by SARS-CoV-2 infection. Wild-type Caco-2 cells were used for SARS-CoV-2 infection, considering that endogenous RIG-I and MAVS can be destructed by Nsp5. When antiviral gene expression was analyzed by real-time PCR, we found that the simultaneous expression of RIG-I-Q10E and MAVS-K136R induced significantly higher levels of transcripts of these antiviral genes than those induced by wild-type RIG-I and MAVS in Caco-2 cells (Fig. 6F) and in *Mavs^−/−^* MEFs (Fig. S5E and S5F).

To assess the biological significance of the inhibition of RIG-I and MAVS by SARS-CoV-2, we assessed SARS-CoV-2 replication in these cell lines by real-time PCR for viral RNA abundance and plaque assay for viral titer. Real-time PCR analysis showed that the expression of wild-type RIG-I or MAVS reduced viral S RNA by ∼75%, while the expression of RIG-I-Q10E or MAVS-K136R reduced it by ∼90% (Fig. 6G). However, no additive effect was observed for wild-type RIG-I plus MAVS or for RIG-I-Q10E plus MAVS-K136R. When RNA-dependent RNA polymerase (RdRp) RNA was analyzed, we observed similar results, with the effects of RIG-I-Q10E and MAVS-K136R being much more pronounced than those of their wild-type counterparts. This result was also obtained from MEFs with wild-type RIG-I and MAVS and with RIG-I-Q10E and MAVS-K136R (Fig. S5G to S5I). The reduced viral RNA also translated into lower viral titers by RIG-I-Q10E and MAVS-K136 and to a lesser extent for wild-type RIG-I and/or MAVS (Fig. 6I). Taken together, these results demonstrate that destruction-resistant RIG-I and MAVS more robustly inhibit SARS-CoV-2 replication than their wild-type counterparts.

### A small-molecule inhibitor of Nsp5 restores innate immune activation to impede SARS-CoV-2 replication.

**FIG 7:**
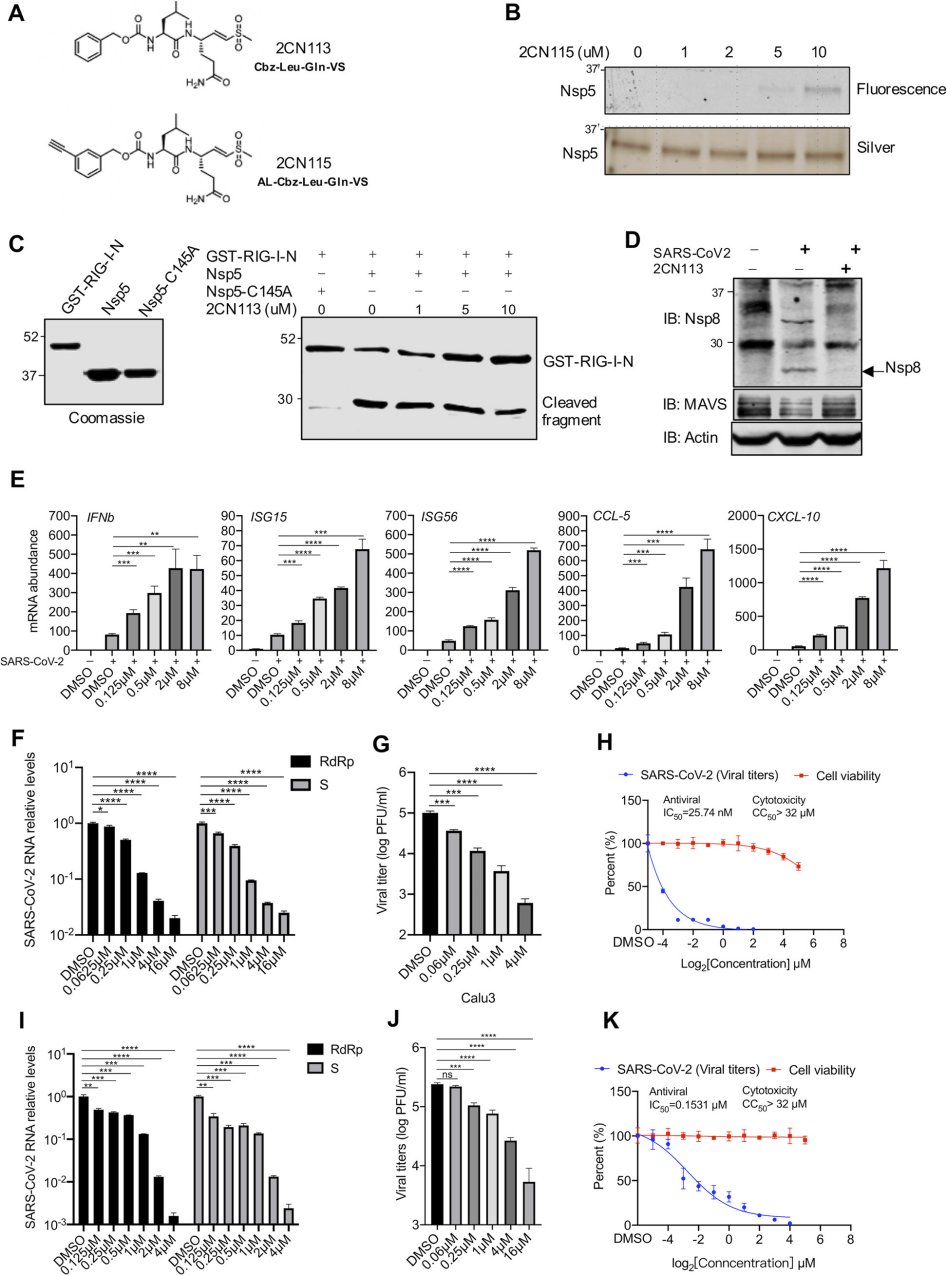
A small-molecule inhibitor of Nsp5 restores innate immune activation and impedes SARS-CoV-2 replication. (A) Structures of 2CN113 and 2CN115. (B) Nsp5-Step-expressing 293T cells were treated with 2CN115 at the indicated doses for 2 h. Nsp5-Step was purified and subjected to binding analysis by in-gel fluorescence imaging and silver staining. (C) The effect of 2CN113 on *in vitro* GST–RIG-I-N cleavage by Nsp5 was analyzed with all proteins purified from E. coli by immunoblotting. (D) HeLa cells stably expressing hACE2 were treated with 2CN113 and infected with SARS-CoV-2 (MOI = 0.1). The effect of 2CN113 on SARS-CoV-2 Nsp8 processing was analyzed by immunoblotting with the indicated antibodies. (E) Calu-3 cells were treated with 2CN113 and infected with SARS-CoV-2 (MOI = 1). The mRNA abundance of antiviral genes was determined by real-time PCR at 72 h after SARS-CoV-2 infection. (F, G) Calu-3 cells were treated with 2CN113 and infected with SARS-CoV-2 (MOI = 0.1). The effect of 2CN113 on the SARS-CoV-2 RNA abundance (F) and viral titer (G) was determined at 72 h after SARS-CoV-2 infection by real-time PCR analysis of total RNA and plaque assay of the medium, respectively. S, SARS-CoV-2 S protein. (I, J) Vero cells stably expressing hACE2 were treated with 2CN113 and infected with SARS-CoV-2 (MOI = 0.001). The effect of 2CN113 on SARS-CoV-2 RNA abundance (I) and viral titer (J) was determined at 24 h after SARS-CoV-2 infection by real-time PCR analysis of total RNA and plaque assay of the medium, respectively. (H, K) The IC_50_ of 2CN113 on SARS-CoV-2 replication by plaque assay and the CC_50_ of 2CN113 on Calu-3 (H) and Vero (K) cells were determined and plotted. Data are means ± SD. Significance was calculated using Student’s two-tailed, unpaired *t* test. *, *P < *0.05; **, *P < *0.01; ***, *P < *0.001; ****, *P < *0.0001; ns, nonsignificant. See also Fig. S6.

In addition to antagonizing the innate immune defense, Nsp5 is a cysteine protease that processes most SARS-CoV-2 nonstructural proteins to yield their functional entities, offering a viable target for antiviral inhibition. Thus, we synthesized two small-molecule inhibitors that contain an electrophilic warhead, vinyl sulfone, to covalently target the catalytic residue of cysteine proteases (Fig. 7A). The vinyl sulfone 2CN115, containing a clickable alkyne tag, was shown to covalently engage Nsp5 in an in-gel fluorescence assay (Fig. 7B). A reporter assay showed that 2CN113 restored IFN induction in the presence of Nsp5 when cells were transfected with RIG-I-N (Fig. 6SA); thus, we further characterized 2CN113 as an inhibitor of Nsp5. When 293T cells were treated with 2CN113, RIG-I protein was restored at 2 μM and, to a much lesser extent, at 0.08 μM and 0.4 μM (Fig. S6B). Similarly, 2CN113 also restored MAVS protein in the presence of Nsp5 (Fig. S6C). Furthermore, 2CN113 reduced Nsp5-mediated RIG-I cleavage in a dose-dependent manner when analyzed by an *in vitro* cleavage assay (Fig. 7C). Considering that Nsp5 is also required for the processing of SARS-CoV-2 nonstructural proteins, we analyzed Nsp8 in SARS-CoV-2-infected cells with 2CN113 treatment. As shown in Fig. 7D, treatment with 2CN113 abolished the production of Nsp8 and an intermediate containing Nsp8 that matches the size of the Nsp7-Nsp8 fusion. When innate immune gene expression was examined in Calu-3 cells infected with SARS-CoV-2, we found that 2CN113 increased the expression of *IFNb*, *ISG15*, *ISG56*, *CCL5*, and *CCL10* in a dose-dependent manner (Fig. 7E). A similar effect of 2CN113 was observed on the expression of ISGs, such as *IFNb*, *ISG15*, and *ISG56*, in SARS-CoV-2-infected Caco-2 cells (Fig. S6D). Conversely, 2CN113 reduced viral RNA transcripts, such as RdRp and Nsp12, in a dose-dependent manner in Calu-3, Caco-2, and Vero cells infected with SARS-CoV-2 (Fig. 7F; Fig. S6E and S7I). Consistently with this result, 2CN113 also potently reduced infectious virions of SARS-CoV-2 in the medium (Fig. 7G and J and Fig. S6F). Specifically, infectious SARS-CoV-2 in the medium was ∼100- and 500-fold lower in Calu-3 cells treated with 2CN113 at 1 and 4 μM, respectively, than in the vehicle-treated groups. To determine whether the inhibition of SARS-CoV-2 replication was due to the cytotoxicity of 2CN113, we assessed the 50% cytotoxic concentration (CC_50_) or 50% inhibitory concentration (IC_50_) in Caco-2 cells without and with SARS-CoV-2 infection, respectively. This analysis showed that the CC_50_s of 2CN113 on Calu-3 and Caco-2 cells were approximately ∼200-fold greater than their IC_50_s of SARS-CoV-2 replication, indicating that the antiviral activity is unlikely to be due to its cytotoxicity (Fig. 7H and Fig. S6G). Similarly, the CC_50_ of 2CN113 on Vero cells was also ∼200-fold higher than the IC_50_ of SARS-CoV-2 replication (Fig. 7K). These results collectively show that 2CN113 inhibits SARS-CoV-2 replication via Nsp5.

10.1128/mBio.02335-21.6 FIG S6 See also Fig. 8. (A) 293T cells cotransfected with plasmids containing RIG-I-N and GFP or Nsp5 were treated with 2CN113 in increasing concentrations as indicated. WCLs were analyzed by a luciferase reporter assay. (B, C) The effect of 2CN113 on SARS-CoV-2 Nsp5-induced RIG-I cleavage (B) and MAVS degradation (C) was analyzed by immunoblotting with the indicated antibodies. (D) Caco-2 cells were treated with 2CN113 and infected with SARS-CoV-2 (MOI = 1). The mRNA abundances of antiviral genes were determined by real-time PCR at 72 h after SARS-CoV-2 infection. (E, F) Caco-2 cells were treated with 2CN113 and infected with SARS-CoV-2 (MOI = 0.1). The effect of 2CN113 on SARS-CoV-2 RNA abundance (D) and titer (E) was determined at 72 h after SARS-CoV-2 infection by real-time PCR analysis of total RNA and a plaque assay of the medium, respectively. (G) The IC_50_ of 2CN113 on SARS-CoV-2 replication by plaque assay and the CC_50_ of 2CN113 on Caco-2 cells were determined and plotted. Download FIG S6, JPG file, 0.3 MB. Copyright © 2021 Liu et al. 2021 Liu et al. https://creativecommons.org/licenses/by/4.0/ This content is distributed under the terms of the Creative Commons Attribution 4.0 International license.

10.1128/mBio.02335-21.7 FIG S7 See also Materials and Methods. Synthetic route and reaction conditions of 2CN113. Download FIG S7, JPG file, 0.1 MB. Copyright © 2021 Liu et al. 2021 Liu et al. https://creativecommons.org/licenses/by/4.0/ This content is distributed under the terms of the Creative Commons Attribution 4.0 International license.

## DISCUSSION

SARS-CoV-2, the etiological agent for CoV disease 2019 (COVID-19), is a positive, single-stranded RNA virus that recently broke into the human population likely via zoonotic transmission (1, 16). Given that SARS-CoV-2 replicates within the cytoplasm of infected cells, it is conceivable that dsRNA replication intermediates of SARS-CoV-2 can be recognized by cytosolic RNA sensors, such as RIG-I and MDA5 (45, 46). Previous studies have implicated MDA5 in sensing SARS-CoV-2, leaving the function of RIG-I unknown in SARS-CoV-2 infection (47, 48). To probe the role of RIG-I in SARS-CoV-2 infection, we discovered that dsRNA derived from SARS-CoV-2-infected NHBE cells potently activates RIG-I. However, SARS-CoV-2 infection induced a weak inflammatory response, as evidenced by IFN-β induction, in NHBE cells. These observations suggest that SARS-CoV-2 subverts dsRNA-induced innate immune activation. Indeed, a screen utilizing the SARS-CoV-2 expression library identified Nsp5 as a potent inhibitor of IFN induction. Biochemical assays show that Nsp5 targets both RIG-I and MAVS, but not MDA5, for destruction. This result may explain the previous implication of MDA5 in sensing coronavirus infection (47, 48). Specifically, Nsp5 cleaves RIG-I immediately after the Q10 residue, leaving the nearly full-length RIG-I protein incompetent to interact with and activate downstream MAVS. Nsp5 demonstrates proteolytic activity toward RIG-I in cells and *in vitro*, adding RIG-I to the growing list of modulators that include NEMO, STAT2, and other coronavirus nonstructural proteins (49–52). Furthermore, the larger cleaved RIG-I fragment binds dsRNA as competently as wild-type RIG-I, thus exerting a dominant negative effect on the dsRNA-induced immune response. Although N proteins of coronaviruses are proposed to sequester RNA (53), the Nsp5-mediated cleavage of RIG-I offers a conceivably more active means of subverting RIG-I and likely impacts signaling events downstream of other RNA sensors, such as protein kinase R (PKR) (54). Intriguingly, our previous study showed that murine gamma herpesvirus 68 induces the deamidation of residue Q10 to evade the RIG-I-mediated innate immune response (40). In an independent study, Zhijian Chen’s group also demonstrated that the 10 N-terminal amino acids are necessary for RIG-I-mediated IRF3 activation (41). These observations highlight an important element in RIG-I that is targeted by two viral pathogens via distinct mechanisms, thus implying convergent evolution.

In addition to targeting RIG-I, Nsp5 targets MAVS for degradation via the ubiquitin-proteasome pathway. Mechanistically, Nsp5 functions as an E3 ligase that adds ubiquitin to MAVS through K48 linkage. Mutational analysis supports the conclusion that K136 of MAVS serves as the receptor for ubiquitin and that the MAVS-K136R mutant is resistant to Nsp5-mediated degradation. Although Nsp5 is defined as a cysteine protease, the ability of its protease domain to catalyze another type of enzymatic reaction is provocative but not unprecedented. For example, the protease domain of Nsp3, a papain-like protease, is known to perform deubiquitination (55, 56), the reverse reaction of ubiquitination that is catalyzed by Nsp5. It would be interesting to determine how these two viral proteases impact each other in terms of protein ubiquitination status and associated biological processes. Our work describes a new function of Nsp5 in catalyzing protein ubiquitination and degradation, thereby terminating RIG-I-mediated immune signaling. The fact that Nsp5 targets both RIG-I and MAVS, two key signaling molecules in the innate immune detection of cytosolic dsRNA, suggests the importance of this pathway in restricting SARS-CoV-2 replication. Presumably, MAVS degradation induced by Nsp5 likely dampens the MDA5-mediated immune activation that was reported to be responsible for SARS-CoV-2 infection. Indeed, ectopic expression of Nsp5-resistant RIG-I-Q10E and MAVS-K136R partly restored antiviral gene expression, thus reducing SARS-CoV-2 replication. The restoration of antiviral gene expression was likely limited due to the inhibition of downstream steps of the RIG-I–MAVS pathway by other viral proteins. We noted that Nsp5 was previously reported to block IRF3 nuclear translocation and IFN induction (57). However, Nsp5 demonstrates no significant effect on IFN induction by overexpressing TBK-1 or constitutively active IRF3-5D. The apparent discrepancy is not clear and remains to be determined.

In addition to processing host factors such as RIG-I and MAVS, Nsp5 processes several nonstructural proteins of SARS-CoV-2, thus playing essential roles in key processes of viral infection, such as genome replication (58). Here, we report the development of a small-molecule inhibitor that demonstrates the ability to block the Nsp5-mediated destruction of RIG-I and MAVS. Consequently, this small-molecule inhibitor elevated antiviral gene expression and reduced SARS-CoV-2 replication. The antiviral activity of the Nsp5 inhibitor likely also stems from its blockage of the proteolytic cleavage of the majority of SARS-CoV-2 nonstructural proteins, including Nsp4 to Nsp16, in addition to its restoration of innate immune activation. The CC_50_s of the small-molecule inhibitor on Caco-2 and Vero-E6 cells were approximately 200-fold greater than its IC_50_ of SARS-CoV-2 replication in those cells, suggesting that its antiviral activity is not due to nonspecific cytotoxicity. These findings provide a proof of concept for targeting Nsp5 to impede SARS-CoV-2 infection and treat COVID-19.

## MATERIALS AND METHODS

### Antibodies and reagents.

Antibodies against IRF3 (FL-425), GST (Z-5), and RIG-I (H-300) were purchased from Santa Cruz Biotechnology. Antibodies against Flag (M2; Sigma), V5 (A190-220A; Bethyl Group), Strep (688202; BioLegend), MAVS (5-20337; Thermo Scientific), RIG-I (SS1A; Enzo Life Sciences), p-S172 TBK-1 (D52C2; Cell Signaling), TBK-1 (3013S; Cell Signaling), p-S396 IRF3 (4D4G; Cell Signaling), and β-actin (Ab8226; Abcam) were purchased from the indicated suppliers. MG132 (M8699; Sigma), cycloheximide (C4859; Sigma), NH_4_Cl (12125-02-9; Fisher Chemical), and [α-^32^P]ATP (BLU003H250UC; PerkinElmer) were purchased from the indicated suppliers.

### Cell culture.

HEK293T, HCT116, A549, Vero-E6, *Rig-i^−/−^*, and *Mavs^−/−^* mouse embryonic fibroblasts (MEFs) were cultured in Dulbecco’s modified Eagle’s medium (DMEM; HyClone) supplemented with 10% fetal bovine serum (FBS; Gibco), penicillin (100 U/ml), and streptomycin (100 μg/ml). Human colorectal Caco-2 cells were cultured in minimum essential media (MEM) supplemented with 10% FBS and antibiotics. All cell lines were maintained at 37°C in a humidified atmosphere of 5% CO_2_.

### Viruses.

Sendai virus (SeV) was purchased from Charles River. SARS-CoV-2 was propagated in Vero E6 cells. Vero E6 cells were seeded at 1.5 × 10^6^ cells per T25 flask for 12 h. Cells were washed once with FBS-free DMEM before viral infection. After being washed, cells were infected with SARS-CoV-2 at a multiplicity of infection (MOI) of 0.001 in FBS-free DMEM and monitored daily. The supernatant was harvested when 20% of cells demonstrated a cytopathic effect (around 72 h postinfection). The supernatant was centrifuged at 3,000 rpm for 5 min and stored at −80°C, after which the viral titer in the supernatant was determined by a plaque assay using a Vero-E6 monolayer. SARS-CoV-2-related viral propagation, viral infection, and vital titration were performed in a biosafety level 3 (BSL-3) facility at USC.

### Plasmids.

Luciferase reporter plasmids for IFN-β, NF-κB promoters, RIG-I, RIG-I-Q10E, MAVS, TBK-1, and IRF3-5D were described previously (59, 60). A cDNA construct was used to amplify and clone RIG-I-(11–925) into mammalian expression vectors. MAVS-K7A/K10A and MAVS-K136A point mutants were generated by site-directed mutagenesis and confirmed by sequencing. Lentiviral expression plasmids for RIG-I, MAVS, and human ACE2 (hACE2) were generated in the vector pCDH-CMV-EF1-Puro or pCDH-CMV-EF1-Hygro by molecular cloning. An expression library of plasmids containing SARS-CoV-2 genes that were cloned in the pLVX-EF1alpha-2×Strep-IRES-Puro vector was kindly provided by Nevan J. Krogan (UCSF).

### qRT-PCR.

Quantitative real-time PCR (qRT-PCR) was performed as previously described (59). Cells were harvested at various time points after viral infection or transfected with the indicated constructs. Total RNA was extracted using TRIzol reagent (Invitrogen). cDNA was synthesized from 1 μg of total RNA using reverse transcriptase (Invitrogen) according to the manufacturer’s instructions. cDNA was analyzed by qRT-PCR using the qPCR-BIO SyGreen Blue mix Lo-ROX (Genesee Scientific). The gene expression level was calculated by the 2^–ΔΔCt^ method (where Ct is the threshold cycle) using *β-actin* or *GAPDH* as an internal control. The qRT-PCR primers are listed in Table 1.

**TABLE 1:** Real-time PCR primers for human, mouse, and SARS-CoV-2 genes

Species	Gene target	Forward primer sequence	Reverse primer sequence
Humans	*IFNB1*	5′-CTTTCGAAGCCTTTGCTCTG-3′	5′-CAGGAGAGCAATTTGGAGGA-3′
	*ISG15*	5′-GTGGACAAATGCGACGAACCCC-3′	5′-TCGAAGGTCAGCCAGAACAG-3′
	*ISG56*	5′-TCTCAGAGGAGCCTGGCTAA-3′	5′-TGACATCTCAATTGCTCCAG-3′
	*IL-6*	5′-CCAGCTATGAACTCCTTCTC-3′	5′-GCTTGTTCCTCACATCTCTC-3′
	*IL-8*	5′-GGCACAAACTTTCAGAGACAG-3′	5′-ACACAGAGCTGCAGAAATCAGG-3′
	*CCL-5*	5′-CTGCTTTGCCTACATTGCCC-3′	5′-TCGGGTGACAAAGACGACTG-3′
	*CXCL-10*	5′-GTGGCATTCAAGGAGTACCTC-3′	5′-TGATGGCCTTCGATTCTGGATT-3′
	*β-actin*	5′-GTTGTCGACGACGAGCG-3′	5′-GCACAGAGCCTCGCCTT-3′

Mice	*Ifnb1*	5′-CCCTATGGAGATGACGGAGA-3′	5′-CCCAGTGCTGGAGAAATTGT-3′
	*Isg15*	5′-TCCATGACGGTGTCAGAACT-3′	5′-GACCCAGACTGGAAAGGGTA-3′
	*Isg56*	5′-CAAGGCAGGTTTCTGAGGAG-3′	5′-GACCTGGTCACCATCAGCAT-3′
	*β-actin*	5′-ACGGCCAGGTCATCACTATTG-3′	5′-CAAGAAGGAAGGCTGGAAAAGA-3′

SARS-CoV-2	*Nsp1*	5′-ACACGTCCAACTCAGTTTGC	5′-CGAGCATCCGAACGTTTGAT-3′
	*E*	5′-ACTTCTTTTTCTTGCTTTCGTGGT	5′-GCAGCAGTACGCACACAATC-3′
	*N*	5′-GGGGAACTTCTCCTGCTAGAAT	5′-GGGGAACTTCTCCTGCTAGAAT-3′

### Lentivirus-mediated stable cell line construction.

Lentivirus production was performed in HEK293T cells as previously described (61, 62). Briefly, HEK293T cells were cotransfected with packaging plasmids (VSV-G, DR8.9) and the pCDH lentiviral expression vector. At 48 h posttransfection, the supernatant was harvested and filtered. HEK293T, *Rig-i^−/−^*, or *Mavs^−/−^* MEFs and Caco-2, HCT116, and A549 cells were infected with lentivirus-containing medium, supplied with Polybrene (8 μg/ml), and centrifuged at 1,800 rpm for 45 min at 37°C. Cells were maintained in DMEM with 10% FBS and selected at 48 h postinfection with puromycin (1 to 2 μg/ml) or hygromycin (200 μg/ml).

### Dual-luciferase reporter assay.

HEK293T cells were cotransfected with the indicated expression plasmids and a reporter plasmid cocktail containing 50 ng of luciferase reporter plasmids (ISRE-luc, IFN-β-luc, or NF-κB) and 5 ng of the TK-*Renilla* luciferase reporter (control vector) by calcium phosphate precipitation. At 24 h posttransfection, whole-cell lysates (WCLs) were prepared and used for a dual-luciferase assay according to the manufacturer’s instructions (Promega). The activities of firefly luciferase and *Renilla* luciferase were determined by a microplate reader (FLUOstar Omega).

### Co-IP and immunoblotting.

Immunoprecipitation was carried out as previously described (40). Briefly, HEK293T cells stably expressing RIG-I-WT were transfected with the indicated expression plasmids for 36 h. WCLs were prepared using NP-40 buffer (50 mM Tris-HCl, pH 7.4, 150 mM NaCl, 1% NP-40, 5 mM EDTA) supplemented with 20 mM β-glycerophosphate and 1 mM sodium orthovanadate. WCLs were sonicated, centrifuged, and precleared with protein A/G agarose for 1 h. Precleared samples were then incubated with Flag antibody (M2)- or glutathione-conjugated agarose for 4 h at 4°C. The agarose beads were washed extensively, and samples were eluted by boiling them at 95°C for 10 min. Precipitated proteins were analyzed by SDS-polyacrylamide gel electrophoresis and immunoblotting.

All immunoblotting analyses were carried out using the indicated primary antibodies (1:1,000 dilution) and IRDye800/700-conjugated secondary antibodies (1:10,000 dilution; LI-COR). Proteins were visualized with an Odyssey infrared imaging system (LI-COR).

### Protein expression and purification.

HEK293T cells were transfected with plasmids containing Flag-tagged RIG-I and RIG-I-(11–925) for 36 h. WCLs were prepared with Triton X-100 buffer (20 mM Tris, pH 7.5, 150 mM NaCl, 1 mM EDTA, 20 mM β-glycerophosphate, 10% glycerol) supplemented with a protease inhibitor cocktail (Roche). WCLs were sonicated, rotated at 4°C for 20 min, and centrifuged at 12,000 rpm and 4°C for 15 min. The supernatant was filtered and precleared with Sepharose 4B agarose beads (Thermo) at 4°C for 1 h. The precleared samples were incubated with anti-Flag M2 agarose beads at 4°C for 4 h. Agarose beads were washed extensively with lysis buffer. Flag-tagged proteins were eluted with 0.2 mg/ml 3×Flag peptide.

Escherichia coli BL21(DE3) was transformed with pGEX-4T-1 or the pET28 plasmid containing RIG-I-2CARD, Nsp5, or Nsp5-C145A. The expression of recombinant GST-tagged protein was induced by 0.1 mM IPTG (isopropyl-β-d-thiogalactopyranoside) at 20°C. Bacteria were harvested, lysed, and incubated with glutathione-conjugated agarose (GE) for 4 h at 4°C. Agarose beads were washed extensively, and GST-tagged proteins were eluted with 10 mM reduced glutathione.

### Ubiquitination assays.

Cells were lysed in 1% NP-40 buffer (100 μl) supplemented with 1% SDS and denatured at 95°C for 5 min. The cell lysates (100 μl) were diluted with 1 ml lysis buffer, and a portion of cell lysates (200 μl) was saved for immunoblot analysis to detect the expression of target proteins. The rest of the cell lysates (800 μl) were precipitated (Denature-IP) with either anti-Flag beads or anti-MAVS (0.2 to 0.5 μg), followed by protein A/G (20 μl). Precipitated proteins were washed three times and subjected to immunoblotting analysis.

For *in vitro* ubiquitination assay, immunoprecipitated Nsp5-Step, Nsp-C145A-Step, and Flag-MAVS were purified from lysates of E. coli and HEK293T cells, individually. Immunoprecipitates were incubated with 100 ng of E1 (Boston Biochem), 400 ng of E2 (UbcH5b; Boston Biochem), and 2 μg of ubiquitin (Boston Biochem) in reaction buffer (50 mM Tris [pH 7.4], 2 mM MgCl_2_, 4 mM ATP; Boston Biochem) and 1 mM dithiothreitol at 30°C for 2 h, and the reaction was terminated by addition of 4× the sample buffer.

### *In vitro* cleavage assay.

Samples containing recombinant Nsp5 and Nsp5-C145A and its substrate, GST–RIG-I-2CARD, were incubated in Nsp5 cleavage assay buffer {20 mM Tris, pH 7.4, 15 mM NaCl, 0.5 mM TCEP [Tris(2-carboxyethyl)phosphine hydrochloride]} at 37°C for 2 h. Reactions were stopped by the addition of 2× SDS sample buffer and subsequent boiling at 95°C for 10 min, and samples were used for SDS-PAGE and immunoblotting analysis.

### ELISA.

Commercial cytokine enzyme-linked immunosorbent assay (ELISA) kits used in this study were human IFN-β (PBL Assay Science) and human RANTES (R&D Systems). Cytokine levels in the media of cultured cells were determined according to the manufacturer's instructions. Absorbance was determined with FLUOstar Omega (BMG Labtech).

### RNA EMSA.

An RNA electrophoresis mobility shift assay (EMSA) was performed as previously described (63). 5′-ppp-dsRNA (5′-triphosphate dsRNA) and control dsRNA were purchased from InvivoGen, and bottom strands were labeled with [γ-^32^P]ATP by T4 polynucleotide kinase (NEB). Purified RIG-I and RIG-I-(11–925) mutants were incubated with dsRNA at room temperature for 15 min. Binding buffer contained 20 mM Tris-HCl (pH 8.0), 1.5 mM MgCl_2_, and 1.5 mM dithiothreitol (DTT). Unlabeled ppp-dsRNA/control dsRNA was used as a competitor at a 100-fold excess. The reaction mixtures were run on 6% native polyacrylamide gels at a constant voltage of 200 V for 35 min. Gels were dried at 80°C for 2 h and subjected to phosphor-imaging analysis. dsRNA sequences were as follows: (i) labeled 5′-ppp-dsRNA (top strand, 5′-ppp-GCAUGCGACCUCUGUUUGA-3′; bottom strand, 3′-CGUACGCUGGAGACAAACU-5′-^32^P) and (ii) labeled 5′ control dsRNA (top strand, 5′-GCAUGCGACCUCUGUUUGA-3′; bottom strand, 3′-CGUACGCUGGAGACAAACU-5′-^32^P).

### Plaque assay.

For SARS-CoV-2 viral titration (plaque assay), Vero E6 cells seeded in 12-well plates (∼100% cell density) were washed with FBS-free medium and infected with 10-fold-diluted medium containing virion particles. At 1 h postinfection, medium was removed, and overlay medium containing 1% low-melting point agarose and FBS-free 1× DMEM was added. At 72 h postinfection, cells were fixed with 4% paraformaldehyde (PFA) overnight and stained with 0.2% crystal violet. Plaques were counted on a light box.

### Syntheses of 2CN113 and 2CN115.

2CN113 and 2CN115 containing the vinyl sulfone-reactive group were synthesized based on a previously published method, with minor modifications, as illustrated in Fig. S7 (64, 65). Briefly, 9-fluorenylmethoxy carbonyl (Fmoc)-Gln (Trt)-OH was subjected to Weinreb ketone synthesis using *N,O*-dimethyl hydroxylamine hydrochloride to yield the Weinreb amide, called intermediate 1. The newly obtained Weinreb amide 1 was then reduced to an aldehyde-containing intermediate 2 with an excess of LiAlH_4_. Intermediate 3, containing an electrophilic vinyl sulfone group, was formed when reacting aldehyde 2 with diethyl methylsulfonyl methyl phosphonate in the presence of NaH. The final multistep reaction, including Fmoc deprotection and amide coupling with Z-Leu-OH or alkyne-tagged Z-Leu-OH followed by trityl removal, successfully yielded 2CN113 and 2CN115. All of the reagents and solvents were purchased commercially and used without further purification, unless otherwise stated. All anhydrous reactions were carried out under a nitrogen atmosphere. Reactions were monitored by thin-layer chromatography (TLC) on glass TLC plates with silica gel coated with fluorescent indicator F254. UV light and TLC stains, including ninhydrin and 2,4-dinitrophenylhydrazine, were used as visualizing agents. Individual intermediates and final compounds were purified by flash column chromatography using an automated Teledyne CombiFlash system. ^1^H and ^13^C nuclear magnetic resonance (NMR) spectra were obtained on a Varian Mercury 400, 400MR, VNMRS-500, or VNMRS-600 spectrometer at ambient temperature.

### In-gel fluorescence imaging.

HEK293T cells transiently expressing Nsp5-Strep were treated with 2CN115 at the indicated doses at 37°C for 24 h. Following probe treatment, cells were harvested and lysed in NP-40 buffer (50 mM Tris-HCl, pH 7.4, 150 mM NaCl, 1% NP-40, 5 mM EDTA) supplemented with protease inhibitor (5 mg/ml; Roche). WCLs were centrifuged, and the protein-containing lysate was normalized to 1 mg/ml. Strep-tagged Nsp5 was then enriched by incubation with StrepTactin agarose for 20 min at 4°C. The beads were washed and subjected to on-resin CuAAC conjugation to a fluorescent rhodamine dye under normal click chemistry conditions {50 μM 6-carboxytetramethylrhodamine (TAMRA)-azide, 1 mM Tris(2-carboxyethyl)phosphine (TCEP), 200 μM Tris[(1-benzyl-^1^H-1,2,3-triazol-4-yl)methyl]amine (TBTA-HCl), and 1 mM CuSO_4_} for 1 h at room temperature in the dark. Upon completion of the click reaction, beads were washed and bound proteins were eluted by boiling the solutions at 95°C for 10 min. Click-conjugated, enriched proteins were fractionated by SDS-PAGE before visualization at a 532-nm excitation and 610-nm emission on a Typhoon 9400 variable-mode imager (GE Healthcare). After fluorescence scanning, gels were silver stained by standard procedures (Pierce) to track Nsp5 expression levels.

### Antiviral and cytotoxicity assays for 2CN113.

For the antiviral assay, a clinical isolate of SARS-CoV-2 was propagated in Vero E6 cells, and the viral titer was determined as previously described. All infection experiments were performed at BSL-3. Preseeded Calu-3 and Caco-2 cells (5 × 10^5^ cells per well) were pretreated with the indicated concentrations of Nsp5 inhibitors for 2 h, and SARS-CoV-2 was subsequently added (MOI of 0.1) to allow infection for 2 h. Then, the virus-drug mixture was removed, and cells were further cultured with fresh drug-containing medium. Drugs were added every 24 h after viral infection until the end of the experiments. At 24 h, 48 h, and 72 h postinfection, virus-containing medium was collected and the viral titer in the medium was determined by plaque assay.

For Vero cells, preseeded Vero cells (1 × 10^5^ cells per well) were pretreated with the indicated concentrations of Nsp5 inhibitors for 2 h, and SARS-CoV-2 was subsequently added (MOI of 0.001) to allow infection for 2 h. Then, the virus-drug mixture was removed, and cells were further cultured with fresh drug-containing medium. At 24 h postinfection, virus-containing medium was collected, and the viral titer in the medium was determined by plaque assay.

For cytotoxicity assays, Calu-3, Caco-2, and Vero E6 cells were suspended in growth medium in 96-well plates. The next day, appropriate concentrations of Nsp5 inhibitors were added to the medium. After 24 h, the cell viability was measured by cell proliferation kit II (XTT) in accordance with the manufacturer’s instructions.
