# Supplementary figures and images for: SARS-CoV-2 Nsp5 Demonstrates Two Distinct Mechanisms Targeting RIG-I and MAVS To Evade the Innate Immune Response
Source: mBio. 2021 Sep 21;12(5):e02335-21. doi: 10.1128/mBio.02335-21 (PMC8546575; doi:10.1128/mBio.02335-21)

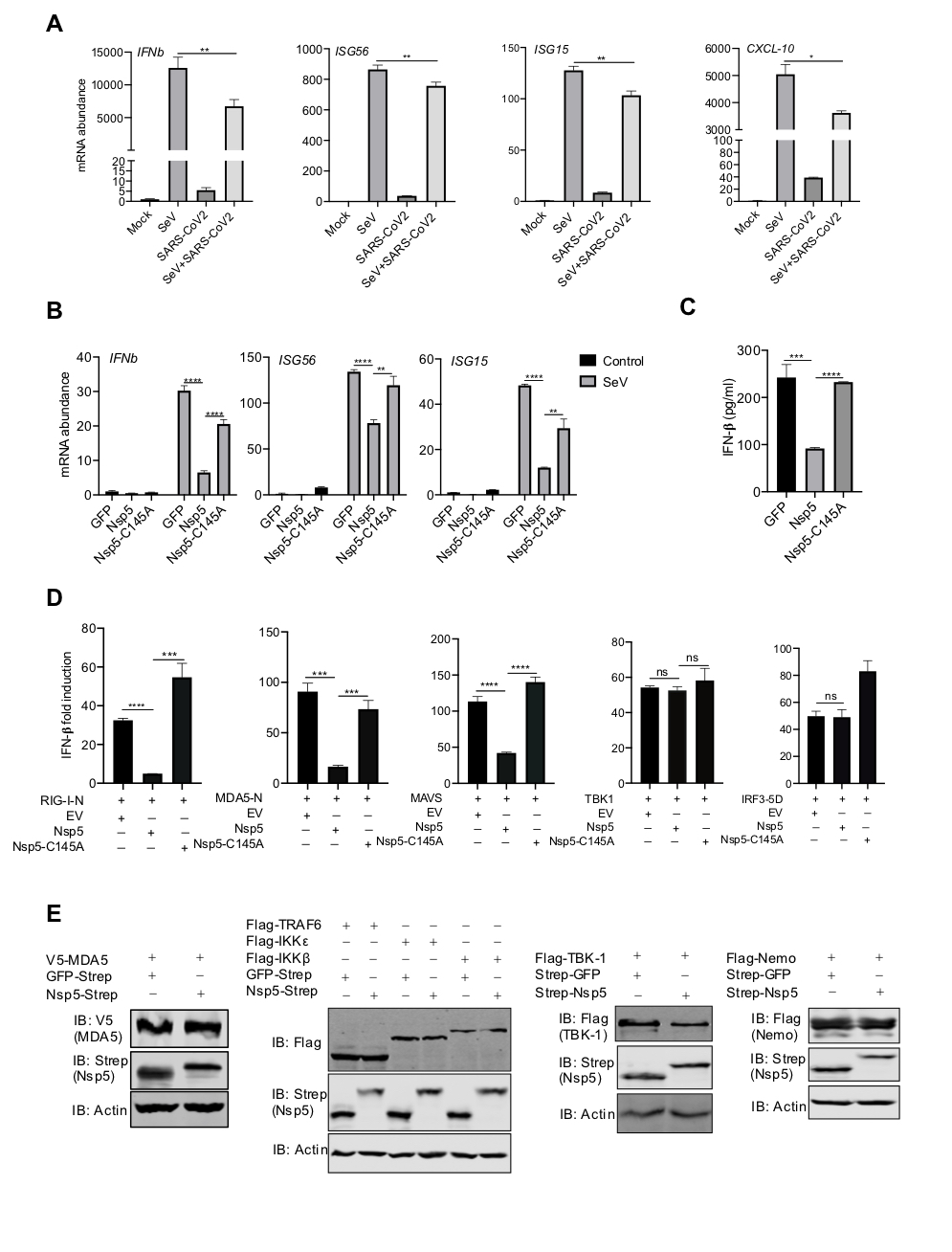

Supplement: FIG S1 [file mbio.02335-21-sf001.jpg]

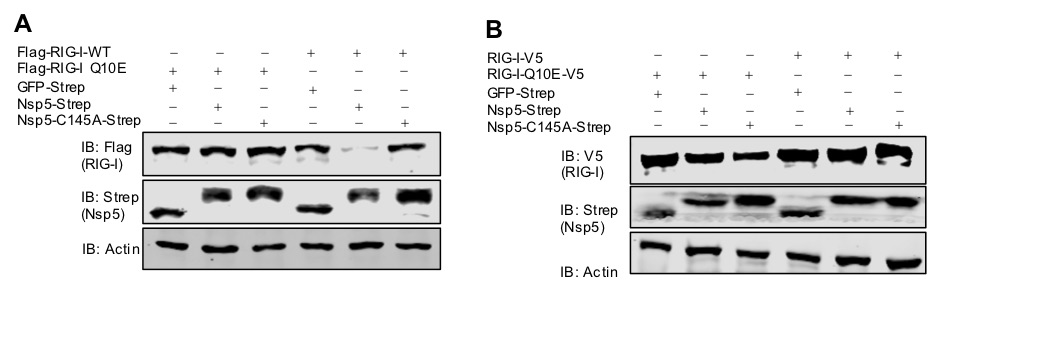

Supplement: FIG S2 [file mbio.02335-21-sf002.jpg]

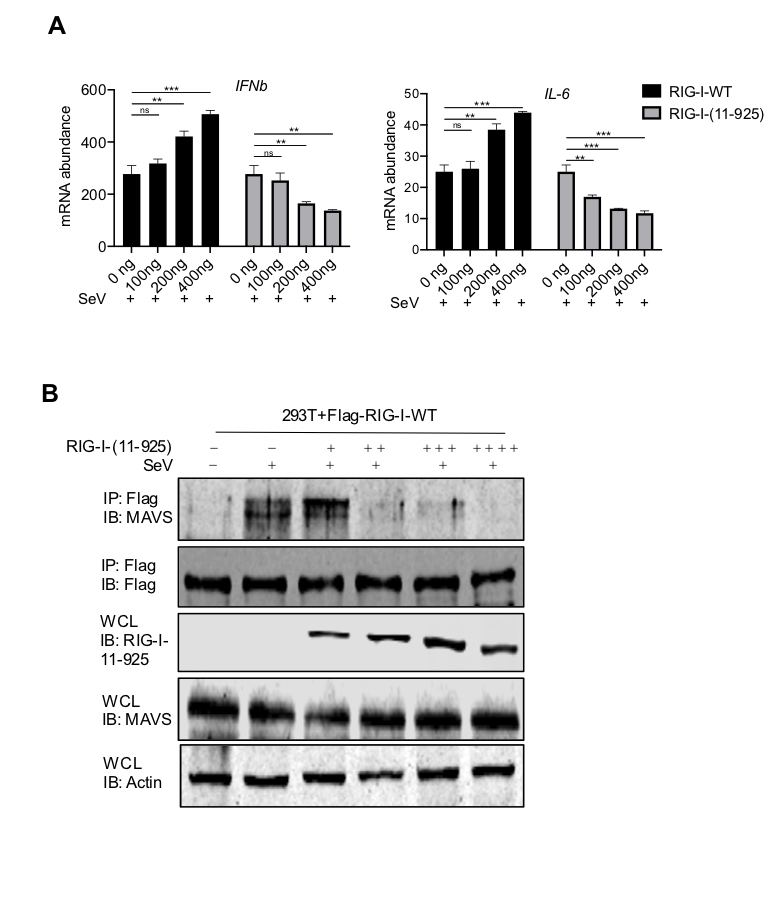

Supplement: FIG S3 [file mbio.02335-21-sf003.jpg]

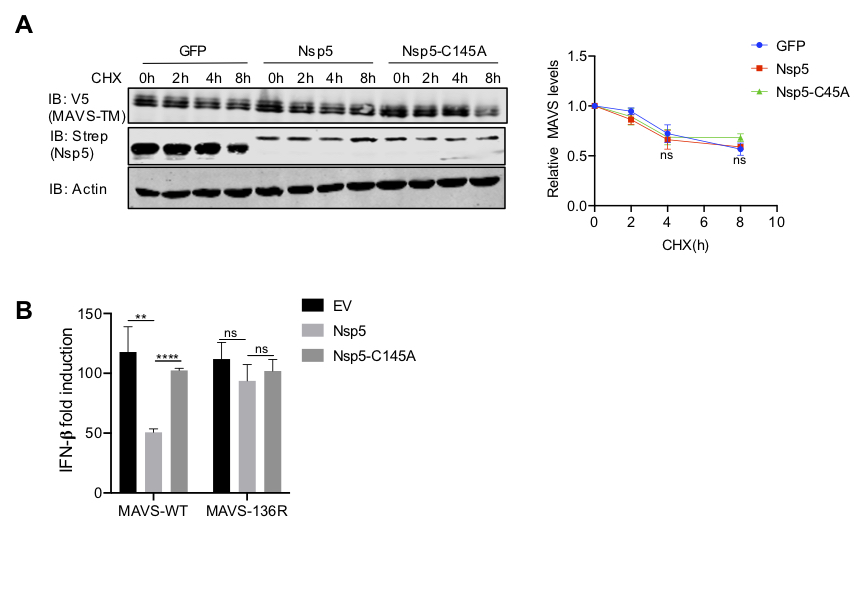

Supplement: FIG S4 [file mbio.02335-21-sf004.jpg]

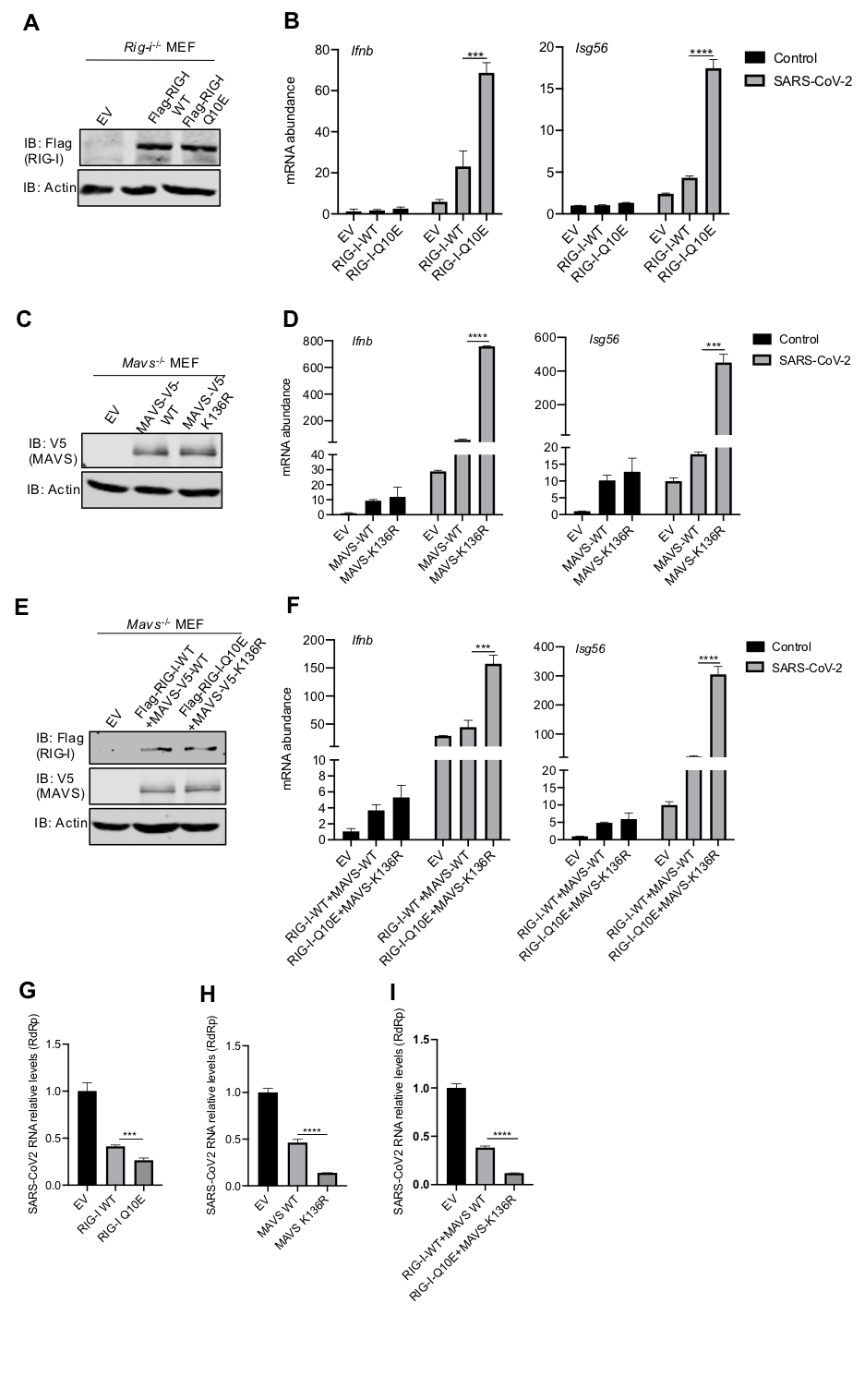

Supplement: FIG S5 [file mbio.02335-21-sf005.jpg]

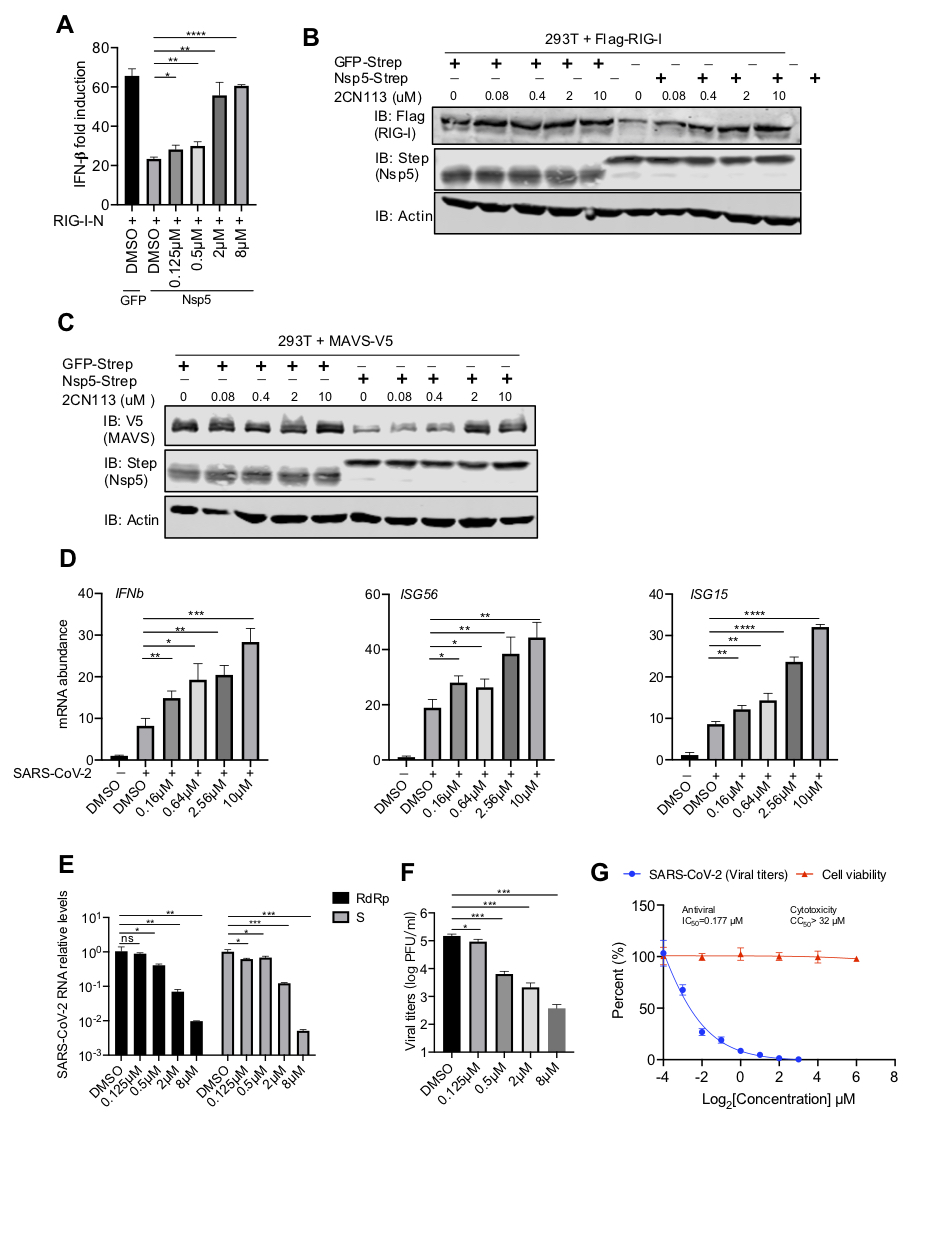

Supplement: FIG S6 [file mbio.02335-21-sf006.jpg]

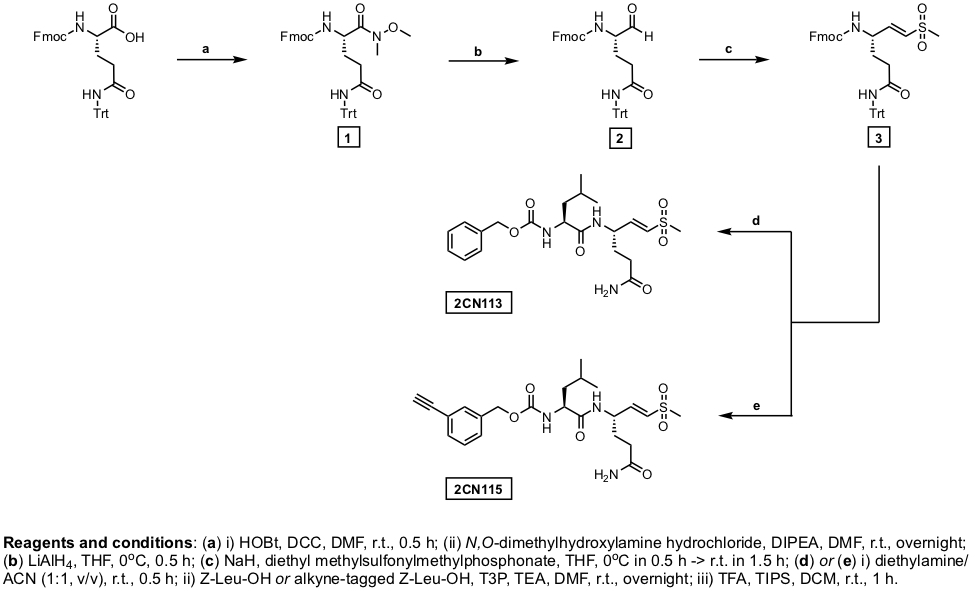

Supplement: FIG S7 [file mbio.02335-21-sf007.jpg]
